# Virtual Interprofessional Telehealth Education: Mixed Methods Evaluation

**DOI:** 10.2196/92077

**Published:** 2026-08-13

**Authors:** Jonathan Liao, Ethan Aminov, Evan Yang, Dale Coffin, Bini John, Nancy Krisch, Kathleen Monahan, Erin Hulfish

**Affiliations:** 1 Renaissance School of Medicine at Stony Brook University, 101 Nicolls Road, Stony Brook, NY, 11794, United States, 1 631-444-2113; 2 Occupational Therapy Program at Stony Brook University, Stony Brook, NY, United States; 3 Stony Brook University School of Nursing Department of Graduate Studies, Stony Brook, NY, United States; 4 Department of Physical Therapy at Stony Brook University, Stony Brook, NY, United States; 5 Stony Brook School of Social Welfare, Stony Brook, NY, United States

**Keywords:** telehealth, telemedicine, interprofessional education, assessment tools, standardized patient simulation

## Abstract

**Background:**

The rapid expansion of telehealth has outpaced formal training in virtual interprofessional collaboration across health professions education. Although telehealth and interprofessional education (IPE) competency frameworks exist, limited evidence informs how longitudinal, virtual IPE curricula prepare learners for team-based telehealth practice.

**Objective:**

This study evaluated a longitudinal virtual interprofessional telehealth course to examine its impact on learners’ perceived telehealth competencies, understanding of interprofessional roles, and readiness for virtual team-based care.

**Methods:**

We conducted a mixed methods evaluation of a 4-week virtual IPE course involving graduate students from medicine, nursing, physical and occupational therapy, and social work. Learners participated in longitudinal standardized patient telehealth encounters, interprofessional debriefing sessions, and asynchronous learning activities. Quantitative outcomes were assessed using a validated postcourse survey. Qualitative data were obtained from open-ended survey responses and analyzed using thematic analysis.

**Results:**

Twenty-eight students completed the course and postcourse evaluation. Quantitative findings demonstrated high self-reported confidence in telehealth communication, ethical practice, interprofessional collaboration, and role clarity, with mean scores ranging from 4.4 (0.7) to 4.8 (0.6) on a 5-point Likert scale. Qualitative analysis identified three core themes: (1) development of interprofessional collaboration skills in virtual settings, (2) growth in telehealth-specific clinical and communication competencies, and (3) perceived value of longitudinal standardized patient encounters with iterative feedback.

**Conclusions:**

A longitudinal, standardized patient–based virtual IPE curriculum was associated with strong self-reported preparedness for interprofessional telehealth practice among health professions students. These findings support the use of structured, longitudinal virtual IPE models to address evolving educational needs in telehealth training.

## Introduction

Prior to the COVID-19 pandemic, telemedicine use in the United States was limited by reimbursement constraints, licensing barriers, and a lack of formal training within health professions education [1]. The pandemic accelerated adoption and revealed the enduring value of telehealth in clinical care, with national data demonstrating sustained use well beyond the initial public health emergency [2]. At the same time, this rapid expansion exposed persistent gaps in clinician preparedness, particularly in communication, ethical practice, and collaboration across professional disciplines in virtual care environments.

As telehealth becomes an enduring component of practice, health professions trainees must be prepared to deliver care effectively in virtual settings. Learners across disciplines frequently report limited confidence in telehealth-specific communication skills and insufficient understanding of other professionals’ roles in remote care [3,4]. National organizations, including the Association of American Medical Colleges and the Interprofessional Education Collaborative, have articulated core telehealth and interprofessional competencies that emphasize team-based practice, role clarity, and patient-centered communication [5,6]. Interprofessional education (IPE) is also supported by constructivist and experiential learning theories, which emphasize learning through collaboration, reflection, and application in authentic practice settings [7,8].

Prior telehealth simulations and telehealth objective structured clinical examinations have demonstrated benefits in improving learners’ confidence, teamwork behaviors, and perceived readiness for telehealth practice [9,10]. Additional mixed methods studies suggest that virtual patients and remote interprofessional learning environments can enhance understanding of professional roles and collaborative care, even among geographically distributed learners [11]. However, much of the existing literature remains limited by short, single-session interventions, discipline-specific training, or cross-sectional evaluation, leaving an important gap in evidence on how interprofessional telehealth competencies develop over time in fully virtual, longitudinal learning environments [12].

To address this gap, a longitudinal virtual interprofessional telehealth course was developed at Stony Brook University for graduate health professions students. The aim of this study was to evaluate the educational impact of this curriculum by examining learners’ perceived telehealth competencies, understanding of interprofessional roles, and readiness for virtual team-based care using a mixed methods approach.

## Methods

### Overall Course

A 4-week iterative virtual IPE course was developed for the summer of 2024 at Stony Brook University in Stony Brook, New York. Students were recruited from the School of Medicine, School of Health Professionals, School of Social Welfare, and School of Nursing. Participation was voluntary, and students were given credit for the course through either elective credit and/or a specialized IPE certificate, based on the requirements of the individual school. Students were eligible for participation if they had completed preclinical rotations and there was no interference with required courses. The course was based on a previously piloted virtual IPE course and revised by faculty members from each of the participating schools (Multimedia Appendices 1 and 2). Objectives covered telehealth core competencies outlined by the Association of American Medical Colleges, as well as the IPE core competencies outlined by the Interprofessional Education Collaborative [5,6].

This 4-week course comprised a hybrid of synchronous and asynchronous activities. Classes met virtually twice a week and covered the specific telehealth learning domains with in-class instruction and asynchronous interactive modules. Each week, the learners participated in small-group virtual standardized patient (SP) sessions with iterative case-based learning scenarios to solidify the core concepts covered during the week. Small groups had a mix of students from each of the 4 schools and were led by a facilitator from one of the health science schools. All facilitators participated in interprofessional training and case development to gain perspective from each of the different professions.

### SP Sessions

The case progressed in a longitudinal timeline over “6 months” for the 4 sessions (Multimedia Appendix 3). Prior to the SP session, students were provided with a “door note” of the case for the next day and the objective of the activities (Multimedia Appendix 4). Small groups met with the same SP over the 4 weeks and followed a structured timeframe. Students began with a 15-minute planning huddle, followed by a 20-minute patient interview conducted by one group while the other observed. Sessions concluded with 10 minutes of self-reflection, 10 minutes of SP feedback, a 25-minute faculty-facilitated debrief, and a brief wrap-up involving all teams (Multimedia Appendix 5). This format ensured that each group had the opportunity to both lead and observe while engaging in interprofessional reflection and feedback. Both the small-group facilitator and the SP completed standardized objective structured clinical examination (OSCE) checklists at the end of each case. (Multimedia Appendices 6 and 7) The facilitator and then the SP reviewed their feedback at the end of each SP session with the students.

### Data Analysis

Learner outcomes were assessed using a validated virtual IPE survey, administered both before and after course completion. The survey was specifically developed to evaluate learners’ confidence, role clarity, and preparedness for interprofessional collaboration in virtual education and telehealth settings. The instrument demonstrated evidence of content validity and reliability during its development and validation process [13]. Paired precourse and postcourse survey items were limited to assessments of familiarity with interprofessional roles; all other survey items assessing telehealth competencies, teamwork, and course perceptions were administered after the course only (Multimedia Appendix 8).

The survey included 5-point Likert scale items assessing learners’ perceived competencies in telehealth communication, ethical practice, interprofessional collaboration, and understanding of professional roles in virtual care. Mean differences were calculated across professional groups to describe perceived changes in role familiarity following course participation. Quantitative survey data were analyzed using descriptive statistics, including means and SDs for Likert scale items along with 1-tailed paired *t* tests.

Qualitative data from open-ended survey responses were analyzed using thematic analysis, following an inductive approach. Two members of the research team independently reviewed all responses and generated initial codes based on recurring concepts related to interprofessional collaboration, telehealth skill development, and longitudinal SP experiences. Codes were iteratively reviewed and refined through discussion, and related codes were grouped into overarching themes. Discrepancies were resolved by consensus to enhance analytic rigor and credibility. Representative quotations were selected to illustrate each theme.

### Ethical Considerations

The Stony Brook University Institutional Review Board reviewed this project and determined that it did not constitute human subjects research and therefore did not require institutional review board approval or exemption (IRB2023-00080). Course evaluation participation was voluntary, data were analyzed in aggregate, and no identifiable student information was reported.

## Results

### Quantitative Findings

Twenty-eight graduate health professions students (n=11, 39.3% medical; n=8, 28.6% physical and occupational therapy; n=3, 10.7% nursing; and n=6, 21.4% social work) completed all 4 SP-based telehealth encounters and the associated precourse and postcourse assessments. Quantitative outcomes were evaluated using paired precourse and postcourse survey data assessing familiarity with other professions, perceived telehealth competencies, and confidence in virtual interprofessional teamwork.

### Familiarity With Interprofessional Roles

Students demonstrated statistically significant improvements in familiarity with the roles and contributions of other health professions following completion of the course (Table 1). Mean increases in familiarity scores ranged from 1.36 to 1.86 points across disciplines, with all paired comparisons reaching statistical significance (*P*<.001). Social work students showed the largest mean increase in familiarity (mean difference 1.86, SD 1.11), while medical students demonstrated the smallest, yet still significant, improvement (mean difference 1.36, SD 1.28).

**Table 1.** Paired-sample *t* tests comparing familiarity with health professions before and after the interprofessional education course (N=28).

Profession	Mean difference (SD)		95% CI	*t* test (*df*)	*P* value
Medicine	1.36 (1.28)		0.86‐1.85	5.60 (27)	<.001
Occupational therapy	1.46 (1.40)		0.92‐2.01	5.53 (27)	<.001
Social work	1.86 (1.11)		1.43‐2.29	8.83 (27)	<.001
Physical therapy	1.50 (0.92)		1.14‐1.86	8.60 (27)	<.001
Nurse practitioner	1.68 (1.22)		1.21‐2.15	7.29 (27)	<.001

### Perceived Telehealth Knowledge and Communication Skills

Postcourse self-assessments demonstrated high perceived familiarity with telehealth-related knowledge and communication skills (Table 2). Mean ratings across items assessing telehealth etiquette, patient-provider communication, motivational interviewing, and interprofessional communication ranged from 4.4 to 4.8 on a 5-point Likert scale. Students reported particularly strong familiarity with effective telehealth communication (mean 4.6, SD 0.6) and understanding of telehealth etiquette (mean 4.6, SD 0.6). Ratings also reflected strong endorsement of interprofessional learning, with students agreeing that working with learners from other disciplines enhances their education and improves quality of care (mean 4.7‐4.8).

**Table 2.** Postcourse self-assessment of telehealth-related knowledge, communication skills, and virtual interprofessional teamwork^a^.

Statement	Score, mean (SD)
Knowledge of telehealth etiquette	4.6 (0.6)
Confidence in using proper patient or provider communication skills in a telehealth setting	4.6 (0.6)
Knowledge of common uses of telemedicine in my profession	4.5 (0.7)
I feel that I can communicate effectively using telehealth	4.6 (0.6)
I have an understanding of how to use motivational interviewing via telehealth to enhance my interview skills with clients/patients	4.5 (0.6)
I am familiar with different formalized communication tools utilized within interprofessional telehealth teams	4.4 (0.7)
Working with students from different disciplines enhances my education	4.7 (0.6)
Health professional students from different disciplines should be educated on establishing virtual collaborative relationships with one another	4.7 (0.6)
During their education, health professional students should be involved in teamwork with students from different disciplines in order to understand their respective roles	4.6 (0.8)
I will be able to share and exchange ideas in a team discussion	4.8 (0.6)
Learning from students from other professions will make me a more effective member of a virtual interprofessional team	4.7 (0.6)
The team approach improves the quality of care to patients	4.8 (0.6)
Developing a virtual patient care plan with other team members avoids errors in delivering care	4.6 (0.7)
The virtual team approach makes the delivery of care more efficient	4.4 (0.9)
The team has a good understanding about their respective responsibilities in virtual settings	4.6 (0.6)
Team members are usually willing to take into account the convenience of individuals when planning their work	4.6 (0.7)
Individuals on the team share similar ideas about how to treat patients	4.5 (0.8)
Team members are willing to discuss individuals’ issues virtually	4.7 (0.6)
Team members cooperate with the way care is organized	4.7 (0.7)
Team members anticipate when they will need others’ help in virtual settings	4.6 (0.8)

a Participants rated their familiarity with each statement after completing the course using a 5-point Likert-type scale, where 1 indicated “very unfamiliar” and 5 indicated “very familiar.”

### Confidence in Virtual Interprofessional Teamwork

Students reported high levels of confidence in their ability to function within virtual interprofessional teams following course completion (Table 3). Mean scores for teamwork-related competencies, including functioning effectively in a virtual interdisciplinary team, identifying discipline-specific contributions to patient care, developing virtual interprofessional care plans, and engaging in shared decision-making, ranged from 4.5 to 4.8. Participants expressed strong confidence in describing their professional roles to team members (mean 4.7, SD 0.6) and in treating team members as colleagues in virtual settings (mean 4.8, SD 0.5).

**Table 3.** Postcourse self-assessment of perceived ability to perform tasks related to virtual interprofessional teamwork and patient-centered care^a^.

Statement	Score, mean (SD)
Function effectively in a virtual interdisciplinary team	4.7 (0.6)
Treat team members as colleagues in virtual settings	4.8 (0.5)
Identify contributions to patient care that different disciplines can offer	4.7 (0.6)
Address clinical issues succinctly in virtual interdisciplinary meetings	4.5 (0.8)
Develop a virtual interdisciplinary care plan	4.6 (0.7)
I can share and exchange ideas in a team discussion	4.6 (0.6)
I have gained an enhanced perception of myself as someone who engages in interprofessional practice	4.6 (0.6)
I feel comfortable speaking out within the team to keep the best interests of the client in mind	4.6 (0.7)
I feel comfortable and describing my professional role to another team member	4.7 (0.6)
I have gained an enhanced awareness of the roles of other professionals on a team	4.6 (0.6)
I have gained an appreciation for the importance of having the client and family as members of the team	4.8 (0.6)
I am comfortable engaging in shared decision-making with the clients	4.6 (0.7)
I feel comfortable accepting responsibility delegated to me within a team	4.7 (0.6)

a Participants rated their perceived ability, comfort, and agreement with each statement after completing the course using a 5-point Likert-type scale, where 1 indicated “very unfamiliar” and 5 indicated “very familiar.”

### Postcourse Assessment of Course Design

Postcourse responses indicated strong learner endorsement of the course design and the educational value of SP-based, longitudinal interprofessional telehealth activities. Students reported that repeated telehealth encounters with SPs enhanced their ability to communicate effectively with other health care professionals in virtual settings and improved their understanding of interprofessional collaboration. The longitudinal, case-based structure was consistently rated as a valuable component of learning, with participants noting increased awareness of patient-specific challenges in telehealth encounters and a clearer understanding of professional roles and responsibilities within a virtual care team.

### Qualitative Findings

Qualitative data from open-ended survey responses were analyzed using thematic analysis, following an inductive approach. Two members of the research team independently reviewed all responses and generated initial codes based on recurring concepts related to interprofessional collaboration, telehealth skill development, and longitudinal SP experiences. Codes were iteratively reviewed and refined through discussion, and related codes were grouped into overarching themes. Discrepancies were resolved by consensus to enhance analytic rigor and credibility. Representative quotations were selected to illustrate each theme (Table 4).

**Table 4.** Thematic summary of students’ comments regarding the interprofessional telehealth course.

Course theme	Comments	Summary evaluation
Interdisciplinary teamwork	“I really liked the disciplines that were brought together for this class. I liked learning from each other and being able to see how all aspects of a person’s life can be addressed in one Telehealth appointment. I thought that the group I was a part of was respectful towards each other and worked well as a team.” “These sessions were very valuable in learning collaboration skills between professions and how to organize our plans and what our focuses would be and how to work together as a group.”	Students felt they learned more about what it means to work as a team in an interdisciplinary telehealth setting.
Telehealth skills	“[There were] noticeable differences in myself (and team’s) ability to interview a pt virtually and do so in an organized and cohesive way for the pt. Could only be done via a longitudinal case over several sessions.” “I feel that I will be a better clinician, especially over telehealth.”	Students felt they were able to hone telehealth interviewing, physical exam, and clinical management skills.
Longitudinal standardized patient care	“Having the standardized patient was a great way to work on skills and have it be a real scenario and getting to see someone that we would be treating and hear the effects their conditions have on them. I really appreciated getting to hear the feedback from our patient each week since it helped us focus on areas in the next session to hit all our goals as well as taking the patients perspective and incorporating it into our goals of focus.” “The standardized patient settings had a massive benefit for my development both interprofessionally and individually as a future clinician.”	Students appreciated the use of a longitudinal case series as it provided a taste of real-life longitudinal patient care and allowed for improvement from session to session.

## Discussion

### Principal Findings

This mixed methods evaluation examined a longitudinal, virtual interprofessional telehealth curriculum incorporating SP encounters. Findings demonstrated statistically significant improvements in learners’ self-reported familiarity with interprofessional roles following course participation, along with high postcourse confidence in telehealth communication, virtual teamwork, and ethical practice. Qualitative findings reinforced these results, with learners describing enhanced collaboration; a clearer understanding of professional roles; and skill development through repeated, team-based telehealth encounters. Together, the quantitative and qualitative data suggest that structured, longitudinal virtual interprofessional experiences may support learner preparedness for collaborative telehealth practice.

These findings align with the study aim of evaluating learners’ perceived telehealth competencies, role understanding, and readiness for virtual team-based care. Improvements in role familiarity were based on paired precourse and postcourse self-assessments, allowing for within-participant comparison while avoiding causal inference. High postcourse ratings across telehealth knowledge and teamwork domains further suggest that learners felt prepared to engage in virtual interprofessional settings. Qualitative themes provided contextual insight into these outcomes, highlighting the importance of continuity, shared responsibility, and iterative feedback in fostering collaboration over time.

Interpretation of these findings is supported by constructivist and experiential learning theories, as learners engaged in social, team-based problem solving and repeated cycles of experience, reflection, and application through longitudinal SP encounters and facilitated debriefing. Consistent with prior telehealth IPE literature, learners reported high confidence in communication and teamwork; however, this study extends existing work by demonstrating the perceived value of a fully virtual, longitudinal interprofessional model involving multiple health professions learners [9-12].

### Limitations

This study has several limitations. The sample size was small and drawn from a single institution, limiting generalizability. This was mainly due to challenges stemming from recruitment. As this was not a required course, students were not given protected time and were unable to participate due to scheduling conflicts across programs. Additionally, the lack of formal academic credit in several schools may have discouraged enrollment and reduced the final sample size. Although paired self-assessment data were used to evaluate changes in role familiarity, other outcomes relied on postcourse self-report and may be subject to response bias. Objective assessments of telehealth performance or patient outcomes were not included.

### Conclusions

As telehealth becomes an increasingly routine mode of health care delivery, findings from this study suggest that longitudinal virtual IPE models may offer a scalable approach to preparing learners for collaborative practice in remote care settings. The longitudinal, team-based structure, supported by facilitated debriefing and iterative feedback, appears to reinforce role clarity, communication skills, and confidence in virtual collaboration. These findings support the educational value of structured, longitudinal virtual IPE models in telehealth training and provide a foundation for future research incorporating objective performance measures, broader disciplinary participation, and multi-institutional implementation.

## Supplementary material

10.2196/92077 Multimedia Appendix 1 Syllabus of the course.

10.2196/92077 Multimedia Appendix 2 Main objectives of each scenario.

10.2196/92077 Multimedia Appendix 3 Standardized patient scenarios.

10.2196/92077 Multimedia Appendix 4 Door note instructions for students.

10.2196/92077 Multimedia Appendix 5 Timeline for interprofessional student teams.

10.2196/92077 Multimedia Appendix 6 Standardized patient checklist.

10.2196/92077 Multimedia Appendix 7 Session evaluation forms.

10.2196/92077 Multimedia Appendix 8 Virtual interprofessional education survey.

## Abbreviations

IPE: interprofessional education
OSCE: objective structured clinical examination
SP: standardized patient
