# Supplementary material for: Virtual Interprofessional Telehealth Education: Mixed Methods Evaluation
Source: JMIR Form Res. 2026 Aug 13;10:e92077. doi: 10.2196/92077 (PMC13472524; doi:10.2196/92077)
Supplement: Supplementary file 1 [file formative-v10-e92077-s008.docx]

Personal identifier code: first initial of mother's maiden name, first initial of street where you grew up and first initial of birth month (ex Levine, Bellview, March = LBM)

Name of Professional Program

- ○ MD
- ○ SSW
- ○ SHP
- ○ SON

To which gender do you most closely identify

- ○ Male
- ○ Female
- ○ Transgender
- ○ Non-binary / non-conforming
- ○ Prefer not to say

Please indicate your primary ethnicity

- ○ Not of Hispanic, Latino or Spanish origin
- ○ Mexican, Mexican Am., Chicano
- ○ Puerto Rican
- ○ Cuban
- ○ Other Hispanic, Latino, or Spanish origin
- ○ Prefer not to say

Indicate your primary race

- ○ Black or African American
- ○ White
- ○ American Indian or Alaskan Native
- ○ Chinese
- ○ Filipino
- ○ Asian Indian
- ○ Vietnamese
- ○ Korean
- ○ Japanese
- ○ Samoan
- ○ Chamorro
- ○ Native Hawaiian/Other Pacific Islander
- ○ Other
- ○ Prefer not to say

Your age

- ○ 21 and below
- ○ 22 - 27
- ○ 28 - 33
- ○ 34 - 39
- ○ 40 - 45
- ○ 46 - 51
- ○ 52 - 57
- ○ 58 - 63
- ○ 64 and up

Please indicate the choice below that best describes the amount of experience you have had with using telemedicine/telehealth prior to the course

- ○ None
- ○ Professional occasional use
- ○ Professional moderate use
- ○ Professional extensive use
- ○ Personal use

Please rate yourself on each of the following knowledge and skills regarding Telehealth using a five-point scale where 5 indicates very familiar and 1 indicates very unfamiliar.

	5 (Very Familiar)	4 (Familiar)	3 (Somewhat Familiar)	2 (Unfamiliar)	1 (Very Unfamiliar)
Knowledge of Telehealth etiquette.	○	○	○	○	○
Confidence in using proper patient/provider communication skills in a Telehealth setting.	○	○	○	○	○
Knowledge of common uses of Telemedicine in my profession.	○	○	○	○	○

Please respond to each question using a five-point scale from Strongly Agree to Strongly Disagree. Choose the one option that you feel best fits your experience/indicates the degree to which you currently hold or display each of the beliefs, behaviors and attitudes that are described.

	Strongly agree	Somewhat agree	Neither agree nor disagree	Somewhat disagree	Strongly disagree
I feel that I can communicate effectively using Telehealth.	○	○	○	○	○
I have an understanding of how to use Motivational Interviewing to enhance my interview skills with clients/patients.	○	○	○	○	○
I am familiar with different formalized communication tools utilized within interprofessional Telehealth teams.	○	○	○	○	○
Working with students from different disciplines enhances my education.	○	○	○	○	○
Health professional students from different disciplines should be educated on establishing virtual collaborative relationships with one another.	○	○	○	○	○
During their education, health professional students should be involved in teamwork with students from different disciplines in order to understand their respective roles.	○	○	○	○	○
I will be able to share and exchange ideas in a team discussion.	○	○	○	○	○
Learning from students from other professions will make me a more effective member of an Interprofessional team.	○	○	○	○	○
The team approach improves the quality of care to patients.	○	○	○	○	○
Developing a virtual patient care plan with other team members avoids errors in delivering care.	○	○	○	○	○
The virtual team approach makes the delivery of care more efficient.	○	○	○	○	○
The team has a good understanding about their respective responsibilities in virtual settings.	○	○	○	○	○
Team members are usually willing to take into account the convenience of individuals when planning their work.	○	○	○	○	○
Individuals on the team share similar ideas about how to treat patients.	○	○	○	○	○
Team members are willing to discuss individuals' issues virtually.	○	○	○	○	○
Team members cooperate with the way care is organized.	○	○	○	○	○
Team members anticipate when they will need others' help in virtual settings.	○	○	○	○	○

Please rate your ability to carry out each of the following tasks using a five-point scale where 5 indicates very familiar and 1 indicates very unfamiliar.

	5 (Very Familiar)	4 (Familiar)	3 (Somewhat Familiar)	2 (Unfamiliar)	1 (Very Unfamiliar)
Function effectively in a virtual interdisciplinary team.	○	○	○	○	○
Treat team members as colleagues in virtual settings.	○	○	○	○	○
Identify contributions to patient care that different disciplines can offer.	○	○	○	○	○
Address clinical issues succinctly in virtual interdisciplinary meetings.	○	○	○	○	○
Develop a virtual interdisciplinary care plan.	○	○	○	○	○
