# Supplementary material for: Virtual Interprofessional Telehealth Education: Mixed Methods Evaluation
Source: JMIR Form Res. 2026 Aug 13;10:e92077. doi: 10.2196/92077 (PMC13472524; doi:10.2196/92077)
Supplement: Supplementary file 1 [file formative-v10-e92077-s001.docx]

| **Interprofessional Education (IPE) & Telehealth Syllabus**  **June/3/2024 to June/28/2024** | | | | | |
| --- | --- | --- | --- | --- | --- |
| **Week 1: Module 1: Overview of Telehealth IPE Teamwork and User Safety** (Facilitator Dr. Erin Hulfish & Dr. Bini John) | | | | | |
| **Week** | **Topic/Objectives** | **Content Available** | **Educational Activities** | **Lead& Presenters** | **To-Do list** |
| Day 1  6/4/2024  4:30 pm to 6:30 pm  (45min)  Day 1  (45min)  Day 1  (15 min) | *Overview of Telehealth IPE and User Safety*   - Welcome slide. - Telehealth Competencies at a glance - IPE Core Competencies at a glance - What you will get out of taking these class   ***Domain I: Patient Safety and Appropriate Use of Telehealth***   - AAMC competency - *Chapter 1*: Who are the users? - When and why to use telehealth. - How to assess patient readiness. - Patient safety and assess risk, during telehealth. - Practice readiness - End-user readiness - *Chapter 2:* The uses, limitations, and benefits of telehealth — that is, the use of electronic communications technology to provide care at a distance. - *Chapter 3*: Determine patient and caregiver access to technology to incorporate telehealth into patient care during real or simulated encounters. - Access to and comfort with technology.   The roles and responsibilities of team members in telehealth encounters regardless of modality.  **Introduction to Standardized Patient Session and JAM Tool** | *Module 1: Overview Intro to Telehealth IPE*   - PowerPoint slide - Detailed script for PowerPoint slide   *Module 1: Patient Safety & Appropriate Use of Telehealth Domain I:*   - 23 PowerPoint slide - Detailed script for PowerPoint slide | - Overview of course - Introduction of Facilitators - Icebreakers, and other small group activities that utilize telehealth concepts, competencies, and communication models. - Group discussion/ Quiz/Knowledge Assessment - Group discussion/ Quiz/Knowledge Assessment - Case studies - Self-study-Module 5: Technology for Telehealth - Large Group discussion on SP preparedness and JAM Tool | Dr. Erin Hulfish  Dr. Bini John  Dr. Erin Hulfish |  |
| Day 2  6/6/2024  4:30-6:30 pm | - Standardized Patient | - Detailed script | - Case studies - Group discussion - Submit Weekly reflection (300 words) by Friday midnight | Dr. Erin Hulfish, Dr. Bini John, Kathleen Monahan, Dr. Dale Coffin, Dr. Nancy Jane Krisch |  |
| **Week 2: Module 2: Communication and Therapeutic Relationship via Telehealth** (Facilitator Dr. Erin Hulfish | | | | | |
| Day 1  6/11/2024  4:30 pm to 6:30 pm | *Module 2: Communication and Therapeutic Relationship via Telehealth.*  ***Domain III: Communication via Telehealth***   - AAMC Competency Covered - *Chapter 1:* Effectively communicate telehealth modalities. - *Chapter 2:* Rapport-building with patients - *Chapter 3*: Building therapeutic relationships and environments during telehealth. - Incorporate patients’ social supports and care providers in the telehealthcare plan. - Group discussion/ Quiz/Knowledge Assessment | *Module 2: Communication and Therapeutic Relationship via Telehealth*   - PowerPoint slide. - Detailed script for PowerPoint slide | - *Guest speaker from Alan Alda Center on Communication* - Group discussion | Kathleen Monahan  (Dr. Erin Hulfish, Dr. Bini John, Kathleen Monahan, Dr. Dale Coffin, Dr. Nancy Jane Krisch) |  |
| Day 2  6/13/2024  4:30 pm to 6:30 pm | Standardized Patient | - Detailed script | - Case studies - Group discussion - Submit Weekly reflection (300 words) by Friday midnight | (Dr. Erin Hulfish, Dr. Bini John, Kathleen Monahan, Dr. Dale Coffin, Dr. Nancy Jane Krisch) |  |
| **Week 3:** **Module 3:Equitable access to telehealth and mitigates cultural biases** (Facilitator Dr. Dale Coffin & Dr. Kathleen Monahan)**.** | | | | | |
| Week 3  Day 1  6/18/2024  4:30 pm to 6:30 pm | *Equitable access to telehealth and mitigates cultural biases.*  ***Domain II: Access and Equity in Telehealth***   - AAMC competency - *Chapter 1*:Recognize and mitigate biases during telehealth encounters. - *Chapter 2:* Promote health equity and access to care and advocate for policy change in telehealth to reduce inequities. - *Chapter 3:*Accommodates the patient’s needs, preferences, and potential cultural, social, physical, cognitive, linguistic, and communication barriers to technology use. - Group discussion/ Quiz/Knowledge Assessment | *Equitable access to telehealth and mitigates cultural biases.* | - Group discussion/ Quiz/Knowledge Assessment   Case studies   - Self-study-Module 6: Data Collection and Assessment via Telehealth - Case studies - Group discussion - Submit Weekly reflection (300 words) by Friday midnight | Dale Coffin  Kathy Monahan  (Dr. Erin Hulfish, Dr. Bini John, Kathleen Monahan, Dr. Dale Coffin, Dr. Nancy Jane Krisch) |  |
| Day 2  6/20/2024  4:30 pm to 6:30 pm | Standardized Patient | - Detailed script | - Case studies - Group discussion - Submit Weekly reflection (300 words) by Friday midnight | (Dr. Erin Hulfish, Dr. Bini John, Kathleen Monahan, Dr. Dale Coffin, Dr. Nancy Jane Krisch) |  |
| **Week 4: Module 4: Ethical Practices and Legal Requirements for Telehealth** (Facilitator Dr. Nancy Jane Krisch) | | | | | |
| Day 1  6/25/2024  4:30 pm to 6:30 pm | ***Domain VI: Ethical Practices and Legal Requirements for Telehealth***   - *Chapter 1:* Understand standards to deliver health care via telehealth. - Ethics in telehealth - Ethical principles - Ethics consult - *Chapter 2:*Telehealth Law and Compliance Issues - Fraud and Abuse laws through the lens of delivering health care through telehealth. - Federal, state, and local facility practice requirements - *Chapter 3:*Components of informed consent for the telehealth encounter. - Ethical challenges and professional requirements in telehealth - Conflicts of interest that may arise in the use of telehealth. | *Module 4: Ethical Practices and Legal Requirements for Telehealth*   - PowerPoint slide. - Detailed script for PowerPoint slide. | - Group discussion/ Quiz/Knowledge Assessment/ Case studies - Preparation time for Sim Lab - *Pro-Con Social Justice Debate* - Group discussion | Dr. Nancy Jane Krisch  (Dr. Erin Hulfish, Dr. Bini John, Kathleen Monahan, Dr. Dale Coffin, Dr. Nancy Jane Krisch) |  |
| Day 2  6/27/2024  4:30 pm to 6:30 pm | *Last Sim Lab* | - Detailed script | - Group discussion/ Quiz/Knowledge Assessment/ Case studies - 15 min for intro - 1hr SP (2 groups each 20-25 min one contiguous interview - 1^st^ has questions, then huddle for action plan 10-15 min then 2^nd^ group presents the plan)   15 min small group wrap up 30-45 min debrief.   - Submit Weekly reflection (300 words) by Friday midnight | Dr. Erin Hulfish, Dr. Bini John, Kathleen Monahan, Dr. Dale Coffin, Dr. Nancy Jane Krisch |  |
| **Self-Study for Week 1**  **Module 5:Technology for Telehealth** | | | | | |
| Self-Study  For Week 1 | ***Domain V: Technology for Telehealth***  AAMC competency   - *Chapter 1:* Basic knowledge of the technology needed for telehealth services. - Use, and teach others while using, equipment for the intended service at originating and distant sites. - *Chapter 2:*Incorporate emerging evidence-based technologies into practice. - Strengths and limitations of evolving applications of technology. - *Chapter 3:*Troubleshoot basic technology failures and optimize settings using the technology. | *Module 5:* *Technology for Telehealth*   - PowerPoint slide - Detailed script for PowerPoint slide | Self-Study Week 1  Week 1 Reflection | Dr. Bini John | Self-Study  Do we want them to have a reflection on this in an assignment?  Assign for week 1 work. |
| **Self-Study for Week 2**  **Module 6:   Data Collection, and Assessment for Telehealth** | | | | | |
| Self-Study  For Week 2 | ***Domain IV: Data Collection and Assessment via Telehealth***  AAMC competency   - *Chapter 1:* Obtain and manage clinical information via telehealth. - Ensure High-quality telehealth care. - Obtain and incorporate the telehealth information into the management plan. - *Chapter 2:* Perform a telehealth physical examination and guide the patient and/or tele-presenter. - *Chapter 3:* Incorporate patient-generated telehealth data into the clinical assessment and treatment plan. - Understand data limitations and adapt a patient-centered care plan. | *Module 6: Data Collection and Assessment via Telehealth*   - PowerPoint slide. - Detailed script for PowerPoint slide. | Self-Study Week 2  Week 2 Reflection | Dr. Dale Coffin | Self-Study  Do we want them to have reflection on this in an assignment?  Assign for week 2 work. |
