# Supplementary material for: Virtual Interprofessional Telehealth Education: Mixed Methods Evaluation
Source: JMIR Form Res. 2026 Aug 13;10:e92077. doi: 10.2196/92077 (PMC13472524; doi:10.2196/92077)
Supplement: Supplementary file 2 [file formative-v10-e92077-s002.docx]

**Main Goals**

**Scenario 1:**

The main goals of this interprofessional student team are to follow-up with the patient, to foster access to care, to complete an initial interview and to assess the patient’s understanding of his health situation. The student team is instructed to:

1. Obtain a general background history of why the patient recently presented to the ER.
2. Introduce Frank to the role of the Interprofessional Team and how the team will work together
3. Assess understanding of back and shoulder pain management including exercise, activity modification including possible equipment use, and medications
4. Assess patient’s ability to utilize telehealth, list three barriers and three benefits
5. Provide collaborative care in conducting a patient interview and initial evaluation/plan

**Scenario 2:**

The main goal of this interprofessional student team is to follow-up with the patient to assess his understanding of his commitment to his health via OARS. The healthcare student team is instructed to:

1. Assess Frank’s feelings about about prioritizing his health needs
2. Assess Frank’s understanding of his commitment to addressing his health via OARS.
3. Assess Frank’s understanding of risks of not addressing his health and mental health care
4. Identify a list of Frank’s health priorities (prioritizing the top 1-3)
5. Create a realistic plan with Frank about 1-3 of his health priorities
6. Provide collaborative care in conducting a patient interview and initial evaluation/plan

**Scenario 3:**

The main goal of this interprofessional student team is to identify ethical dilemmas about Frank’s situation, and address 2 of these ethical issues with him. The healthcare student team is instructed to:

1. Review the 1-3 of his health priorities from last week
2. Address ethical issues and prioritizing patient needs.
3. Assess Frank’s awareness of the social issues that he is facing
4. Assess Frank’s understanding of resources that may be available
5. Address 2 ethical dilemmas with Frank (identified by Frank or the team)
6. Provide collaborative care

**Scenario 4:**

The main goal of this interprofessional student team is to:

1. Explain the diagnosis, treatment, and importance of treatment to the patient in plain language.
2. Assess the patient's understanding of his diagnosis (what is the disease)
3. Assess the patient’s understanding of the treatment (role of the medication, how to take it, possible side effects)
4. Assess the patient’s understanding of self-care and nutrition (how and when to check blood sugar, dietary changes to be made, dental evaluation, checking feet, quit smoking, exercising and making follow-up appointments)
5. Assess the patient’s understanding of diabetes and why taking the medication and checking the blood sugar, maintaining a healthy diet and exercise are important
6. Provide collaborative care in conducting a safe patient discharge.
7. Ensure comfortability with using telehealth technology for follow-up
