# Supplementary material for: Virtual Interprofessional Telehealth Education: Mixed Methods Evaluation
Source: JMIR Form Res. 2026 Aug 13;10:e92077. doi: 10.2196/92077 (PMC13472524; doi:10.2196/92077)
Supplement: Supplementary file 3 [file formative-v10-e92077-s003.docx]

**Case Summary**

**Scenario 1:**

Pt. is 48 y.o. Hispanic male being referred to interdisciplinary telehealth visit by the community hospital emergency department after evaluation for back pain as he was identified as “at risk” for multiple health problems and behavioral health needs for himself and his children. He has not been to an established clinic practice in years and has declined in person referrals to social work services, occupational therapy, physical therapy services and home care nursing. Pt. was found to have lumbar muscle strain due to muscle strength and range of motion imbalance as well as poor body mechanics and postural deformities. He also experiences decreased range of motion from a previous injury to his left shoulder. He is aware he has high cholesterol and high blood pressure, both which are poorly controlled and acknowledges that he has mild to moderate depression.

He is motivated to follow the advice of the health care team, but he is feeling overwhelmed about his overall situation, including the new medications he was prescribed and how he is supposed to fit recommendations into his work and family life. His back is feeling a little better, but he is worried about being able to do his job and take care of his youngest sons and father.

Your challenges, as the standardized patient, include:

1. You are overwhelmed about your situation and concerned about your job and family.
2. You are unsure about what you are supposed to be doing about your back.
3. You are unclear about the medications that you are supposed to be taking.
4. To observe the behaviors and communications of individual students through telehealth and the team as a whole while you are performing this case.
5. To accurately recall the behaviors and communications and accurately complete the performance checklist.

The important challenges of this case for interprofessional student team are to:

1. Obtain a general background history of why the patient recently presented to the ER.
2. Introduce Frank to the role of the Interprofessional Team and how the team will work together.
3. Assess understanding of back and shoulder pain management including exercise, activity modification including possible equipment use, and medications.
4. Assess patient’s ability to utilize telehealth, list three barriers and three benefits.
5. Provide collaborative care in conducting a patient interview and initial evaluation/plan.
6. Ensure comfortability with using telehealth technology for follow-up.

To review:

Your main concerns are:

1) Taking care of your job responsibilities and your family.
2) Making sure that your back pain is under control.
3) Getting some answers about your medications.
4) How will this team address your health concerns

The reasons for your low back strain:

1. Sitting a long period in the truck

2. Deconditioning.

3. You have received limited back education via your employer, but you are remotely aware that you could be doing better with your body positioning when you lift things or help your father.

4. You have been compensating for how you do tasks because of your left shoulder pain (you are a little aware of this, but not the full extent).

**OPENING STATEMENT**:

**“They gave me this medication discharge sheet. Honestly it’s been a lot of information over the past several days”**

**APPEARANCE AND BEHAVIOR**:

**Appearance**:

- Neat, wearing street clothes and sitting in front of computer at home.

**Behavior:**

- **You are sitting, occasionally fidgeting because of your concern about possible back pain. Your back is not hurting right now, but you are aware that it begins to get achy when you sit.**
- You are feeling curious about the telehealth option. You hate going to the doctor’s office and are thinking that this might be a good solution for you – who knows, you are willing to give it a chance.
- You like using the computer to surf the internet but you’re not entirely confident. You don’t want to press the wrong buttons to lose the call. Your 18-year-old set up the computer.
- You are overwhelmed with the information you have been given regarding your diagnosis and discharge plan (Medication History and Reconciliation Form)
- You are feeling stressed because you are the primary caregiver for the family including your elderly father.

**Case Props**:

- A copy of your discharge paper: Medication History and Reconciliation Form (Last page of the case).

**HISTORY OF PRESENT ILLNESS**

You presented to the hospital ER two days ago because your back was hurting so badly that you could not drive your truck. You hate asking for help but you had to ask your eldest son to drive you. When they asked you for a number on a pain scale – you said 8/10. “It was kind of like if someone kicked me maybe ten times in the back – I don’t know. I don’t do kick boxing, but it hurt really bad”. You were diagnosed with a lumbar muscle strain, and treated with IV fluids and a muscle relaxant in the ER that immediately made you feel better. Now you are supposed to not overstrain yourself “whatever that means'' and take Ibuprofen. They also referred you for a follow-up with the telehealth interprofessional healthcare team, since you said you would probably not go to an in person follow-up.

**YOUR UNDERSTANDING OF recent ER visit and Lumbar Muscle Strain**:

- You have bad posture.
- You should be careful when you are lifting things or helping people physically.
- You need to take medication to manage discomfort short term.
- You might get help from physical therapy and occupational therapy.
- You need to rethink your diet, exercise and self-care habits.
- You should be taking your other medicines.

**RESPONSE TO OTHER QUESTIONS**

If the student asks you if you are in a space that you feel comfortable speaking about your recent discharge from the emergency room:

**“Yes, I am in my kitchen.”**

If the student asks you where your kids are:

**“My children are out.”**

If the student asks you where your father is:

**“My father is watching TV in another room.”**

If the student asks you to confirm your address:

**“Yes, it is the one that you have listed on the chart 124 Ridgefield in Rocky Point.”**

If the student asks you to confirm your phone number:

**“631-555-1234”**

If the student asks you if you understand your discharge instructions:

**“Not completely. Like I said, it was a lot to take in.”**

If the student asks about your understanding of telehealth and technology (call from nursing for set up):

**“Well, I got a call from the nurse after I left the ER. The nurse asked if I had gotten the telehealth link and if I had a computer. But that was it.”**

If the student asks if they can explain anything again:

**“That would be good.”**

If the student asks you about your diet:

**“I like sugar. I just do. I know I probably have too much salt also.**

If the student asks you about your cooking meals:

**“It’s really hard to cook. Maria did all that stuff.”**

If the student asks you if you have been educated about eating healthy:

**“Yes, but can you tell me something that a real person can actually do?”**

If the student asks you about any knowledge of posture and body mechanics:

**“Oh, I know I’m supposed to face what I am lifting. And bend my knees. Keep things close.”**

If the student asks you about how your back feels:

**“My back hurts when I sit. I’m not sure why.”**

If the student asks what positions are best for your back:

**“Honest? I don’t know.”**

If the student asks what exercises are best for your back:

**“I am really not sure what I can and can’t do.”**

If the student asks what exercises you do:

**“I tried going to the gym because I was trying to lose weight, and then I hurt my shoulder. I guess I overdid it.”**

If the student asks when you exercise:

**“I tried about 6 years ago, and then I hurt my shoulder and stopped going.”**

If the student asks if you if you had your shoulder checked:

**“No. I just never got to it.”**

If the student asks why you did not get shoulder checked:

**“That takes time I don’t have. And not for nothing, I don’t need to have to pay another copay.”**

If the student asks if you are depressed:

**“Yes, I have been down since my wife passed.”**

If the student continues to ask more than two questions about your depression:

**“I can’t talk about this” (shut down conversation)**

If the student asks if you have thoughts of suicide:

**“No. I would never do that.”**

If the student asks about why you haven’t filled your Wellbutrin prescription?
**“How are pills going to help?”**

If the student asks you to show them your medicines:

“**My back does not hurt right now. I’d rather just rest and enjoy it. I’ll show you some other time.”**

If the student asks about tobacco use:

**“Yes, I quit about 10 years ago, but started again after my wife (Maria) passed. It’s just been hard without her.”**

If the student asks how often you smoke:

**“It depends. When I am stressed.”**

If the student asks how much you smoke:

“**Only 2-3 a day. I know I shouldn’t, but it helps at the end of the day.”**

If the student asks you how often you buy cigarettes:

**“Maybe once a month.”**

**PAST MEDICAL HISTORY**

**Medical Conditions**

- High Blood Pressure
- High Cholesterol
- You had been prescribed medication for your blood pressure that you are not taking consistently, any refills are done randomly. You want to manage the high cholesterol with diet changes, but it’s hard. You don’t like cooking. Maria did that stuff. You are thinking that maybe you need to start taking your medications more regularly, but you’re not sure what they are for.
- Prior to starting with the inter-professional group today, you have not seen a regular physician for more than 10 years.
- Left Shoulder Injury: 6 years ago, lifting weights at the gym, also recent pain post slip and fall in shower (you have not had time to get this checked out by a doctor).
- Depression

**Mental Health**

You have been recommended to see a counselor for concern of depression in the past but have not yet seen one in person. You basically feel overwhelmed at caring for both your children and your father by yourself as well as having to be the sole provider for your family.

Additional information if asked:

- You sleep 5 hours a night.
- You find joy in spending time with your children and friends at work.
- You have not wanted to see a counselor for fear of social concerns of behavioral health and have been raised to “tough it out.”
- You acknowledge that you are depressed after your wife’s early death. You are aware that you are depressed, but do not associate this necessarily with her death. You do not fully understand what depression is or how it affects your life. You do not want to talk about it.

**PAST HOSPITALIZATIONS**

None.

**MEDICATIONS**

**Prescription Drugs:**

- Labetalol 25 mg daily - Taken in the AM (for High Blood Pressure diagnosed 10 years ago)
- Hydrochlorothiazide 25mg daily - Taken in the AM (for High Blood Pressure diagnosed 10 years ago)
- Lisinopril 10 mg daily - Taken in the AM (for High Blood pressure diagnosed 10 years ago)
- Wellbutrin for depression but did not fill prescription.

**Over-the-Counter Drugs:**

- Ibuprofen – 200 to 600 mg NEW after leaving hospital, for low back pain and occasional headaches and for pain you associate with day-to-day physical work and “getting older”.

**Illicit/Street Drugs:**

None. **“No, I would never do that!!”**

**SEXUAL HISTORY**

Not sexually active.

**LIFESTYLE/HABITS**

**Occupation:**

You are a food delivery driver. You consider yourself a reliable and good worker. You get along well with your coworkers and enjoy the social interaction. You have good health insurance through your work.

You enjoy your job and your colleagues but spend most of the day on the road in the truck.

You are concerned that the low back pain could affect your ability to drive your truck. **The back pain is not new, and you had been trying to intentionally plan driving to build in standing breaks.** Standing makes you feel better.

When did the back pain 1^st^ start? How long ago?  **“My back pain started a couple of months ago, but got worse over the weekend and ibuprofen wasn’t helping so I went to the ED.”**

What caused it? **“Not sure what caused it, I had tried working out a couple of months ago and maybe it happened then.”**

What was done in the past for it? **“I’ve just taken ibuprofen on and off when I feel it.”**

**Alcohol:**

**“Yes”** You drink “a couple of beers every night”.

**Tobacco:**

**“Yes”** I quit about 10 years ago, but started again after my wife (Maria) passed. It’s just been hard without her.”

If follow up “**Only 2-3 a day. I know I shouldn’t, but it helps at the end of the day”**

When did you 1^st^ start smoking? **“I’ve been smoking since I’m a teenager.”** (you do not recall

age).

How much did you smoke back then? **“2-3 cigarettes a day.”**

**Caffeine:**

**“Yes”** You drink 1 cup of coffee per day in the morning. No tea, cola. Occasional chocolate

bars.

**Diet:**

**“I love my sweets and have been eating them a long time. ”**

You don’t follow any special diet. You frequently snack on candy during the day or grab a quick meal on the road.

If asked to describe your typical eating for the day:

**Breakfast**: **“Something quick – I’ll pick up – usually a bagel or donut with a cup of coffee.”**

**Lunch**: **“Usually get something from the deli or the lunch truck – sandwich, soda, chips.”**

**Afternoon snack: “I like a candy bar midafternoon – I usually need a “pick me up”**

**Dinner**: **“Don’t cook much – most of the time I am too tired to eat after work.”** You are too tired after work and have to come home to take care of your family. Sometimes you will have a frozen meal, more often lately you will snack on chips or candy at home.

You have been told sometime in the past when your wife was alive to eat a balanced diet, less salt and watch the sugar. Maria helped with that, but since she passed it’s been hard and you have been working a lot to help provide for your family.

**Exercise:**

**“No”** You don’t have a regular exercise routine. You are too tired after work to exercise. You were also injured while exercising 6 years ago and still have difficulty raising your left arm over your head. If asked if the injury interferes with your job - you do not do a lot of exercise with your position at work.

**Activities/Hobbies:**

You like to read. No other hobbies as you are too tired after work.

**Sleep:**

- You get up early (6:00 am) to be at work by 8:00 am. You finish working by 5:00 pm and then come home to care for your father and frequently skip dinner. You are in bed early because of the early hour you get up.
- You are slightly tired from the transition back to home after the hospital. You are sleeping 5 -6 hours per night since you have less back pain. This is ‘pretty decent’ for you.

**FAMILY MEDICAL HISTORY**

Your **father** is alive. He is 78 years old. He suffered a stroke 6 months ago and following five days of hospitalization he was discharged for three weeks to a rehabilitation center.  The rehabilitation center discharge plan, which included follow up medical and at-home physical and occupational therapy never happened. Your father requires help moving around the apartment and needs a walker to get around. While he can feed himself he does have limitations and is frequently forgetful to take his medications. You have been trained as an e-helper for his OT needs but your dad resists your care and is determined to do it on his own. Your dad has refused care from his grandchildren and you have not wanted to burden them by asking for help. The older one is in community college and works at night to help contribute and you value his education and don’t want to place too many demands on his time.

Your **mother** is deceased, died in a car accident 15 years ago. No brothers or sisters.

You did not have a close relationship with your **grandparents** because they lived in another city. Your **mother’s father died** of a heart attack at age 60. Your other grandparents are no longer alive and you are unaware of their health history. If asked, you did not get along with your wife’s side of the family, so have no information to offer clinicians with respect to care for your children.

**PERSONAL/FAMILY HISTORY**

- You are originally from Nicaragua and came to the U.S. with your family when you were 5.
- You grew up as an only child in a working lower economic class family in Queens.
- You have a high school education and have worked all your life as a truck driver.
- You met and married your now deceased wife, Maria, in Queens and then moved to Rocky Point. You were married for 22 years until she died 5 years ago after a 2-year battle with breast cancer.
- You live with your three sons: 18, 16 and 12yo with whom you are very proud. They are in good physical health, and all did well in school until your wife passed. You sense that all of them are “broke without their mother”. The children were 13, 11, and 7 when your wife died. The 18 year old successfully graduated high school, takes class at a community college and works at an auto body shop. When not working he is often with his girlfriend. You like the girlfriend. The 16 year old had been working at a pizzeria until recently has been skipping work and school. You were notified a couple of months ago that he could be in danger of repeating the 11^th^ grade. The 12 year old is very attached to you and your father. He is missing your oldest son.
- Your elderly father has lived with the family for about 15 years.
- You have lived in the same apartment for 30 years.
- The area is a socioeconomically disadvantaged urban neighborhood, but you feel safe and have known your neighbors for years.
- You have struggled with food insecurity in the past. Your wife did the shopping and cooking. With her gone, you have relied more on fast food and candy snacks for yourself to be able to provide for your children. The family is doing a little better financially since your 18 and 16 year old started contributing financially, often taking it upon themselves to arrange their own meals.

**SCENARIO DEVELOPMENT**

**End of the encounter:**

If the inter-professional student team says anything indicating that they have heard your concerns (i.e., they empathize) OR if they discuss ways, to address your concerns, you are agreeable to any suggestions.

You agree to a follow-up **telehealth** appointment if that is suggested. **You agree to upload any information or monitoring, if suggested.** If the inter-professional student team has done a good job of convincing you how important it is for you to follow recommendations, you agree to do what is suggested. You would also agree to see other clinicians via telehealth if recommended.

**ADDITIONAL QUESTIONS YOU MAY ASK ( if needed during the encounter)**

****You do not need to ask all of these questions.**

1. If I stop driving, will my back pain go away?
2. Will I have it for the rest of my life?
3. Is the back pain related to my shoulder?
4. What should I do to keep myself from getting worse?
5. Should I change my diet?
6. Should I start exercising?
7. If I exercise for my back and shoulder, will that help with my hypertension or my cholesterol?

**SP CHALLENGE QUESTIONS:**

At any time that it feels appropriate/natural, you may ask the student:

**“Why are you so concerned about my dad/kids? I am the one with the back pain?”**

If no one addresses or comments on the fact that you are fidgeting or repositioning in chair you will ask:

**“What am I supposed to do so I can sit without my back hurting?”**

If you are asked any questions, you aren’t aware of or if learners refer to any conversations they had with you in the past, you will respond:

**“I don’t remember talking about that. I guess I’m just overwhelmed, I have a lot going on.”**

If any learner asks something that you do not know the answer to, you will respond:

**“I’m really not sure” or “Wow, I haven’t thought of that in a long time. I don’t remember”**

**TIMELINE**

- 10 years ago, diagnosed with hypertension and high cholesterol
- 5 years ago, your wife died
- 6 months ago, your Father (who lives with you) suffered a stroke
- This week you went to the ER with low back pain and were diagnosed with lumbar muscle strain. You were referred for an Interdisciplinary Telehealth Visit by community hospital ER. Identified as high risk for health problems and community needs
- Today you are having a Telehealth visit with the Interdisciplinary Telehealth Team as an option to access healthcare.

**Scenario 2:**

Pt. is a 48 year old Hispanic male referred 3 months ago to interdisciplinary telehealth by the community hospital emergency department after evaluation for back pain as he was identified as “at risk” for multiple health problems and behavioral health needs for himself and his children. He had not been to an established clinic practice in years and had declined in person referrals to social work services, occupational therapy, physical therapy services and home care nursing. At the ER 3 months ago, Mr. Rivera was found to have lumbar muscle strain due to muscle strength and range of motion imbalance as well as poor body mechanics and postural deformities. He also experienced decreased range of motion from a previous injury to his left shoulder. He is aware he has high cholesterol and high blood pressure, both which are poorly controlled and acknowledges that he has mild to moderate depression.

He is motivated to follow the advice of the health care team, but he feels that he does not have much time to take care of his needs. He is tired and at a loss as to how he is supposed to fit health recommendations past and present into his work and family life. He prioritizes helping his father with his father’s telehealth visits, over his own needs. Frank continues to smoke, looks exhausted, admits he isn’t sleeping and only eats only one meal per day (I’m not hungry”). When he does eat, he snacks on unhealthy snacks throughout the day. Frank is also inconsistent in taking his medications for his back and shoulder pain, hypertension and cholesterol. Frank still has not filled the prescription for his anti-depressant. Frank admits that he is not performing exercises prescribed by the PT/OT and states “I just don’t have the time”. When encouraged to add his own self-care to his “to do” list, Frank reports that he can’t do that because he would then feel guilty putting his own needs over his father’s (“I can’t do that. It would kill me to neglect my father”). He also mentions that he doesn’t see his having an ability to change in his situation (“I need to do what I need to do and the rest will take care of itself”). He is avoidant when asked about the children’s health and behavioral health needs.

Your challenges, as the standardized patient, include:

1. You will present as tired, physically, and emotionally (stressed, but not aware of it).
2. Sharing that you know your health is important, but your father and son’s needs are more important to you.
3. Expressing that you do not see a way that he can do anything any differently. (“I need to do what I need to do and the rest will take care of itself”)
4. Avoiding conversation about the needs of his children (and the fact that he is having trouble taking care of them).
5. To observe the behaviors and communications of individual students through telehealth and the team as a whole while you are performing this case; and
6. To accurately recall the behaviors and communications and accurately complete the performance checklist.
7. Identify your health priorities:
   a. Back pain- reducing it
   b. Getting Sleep (reducing stress-only with conversation with the team does he realize stress is related to sleep)
   c. Eating right

The important challenges of this case for interprofessional student team are to:

1. Assess Frank’s feelings about about prioritizing his health needs
2. Assess Frank’s understanding of his commitment to addressing his health via OARS.
3. Assess Frank’s understanding of risks of not addressing his health and mental health care
4. Identify a list of Frank’s health priorities (prioritizing the top 1-3)
5. Create a realistic plan with Frank about 1-3 of his health priorities
6. Provide collaborative care in conducting a patient interview and initial evaluation/plan

To review:

Your main concerns are:

1) You are tired and do not see how you can do anything differently
2) You want to take care of yourself
3) Most important to you is to take care of your father and sons – yourself last

The reasons you are tired include:

1. Work

2. Poor sleep (though unaware that this is due to stress)

3. Poor diet

4. Depression (underlying everything, you miss your wife. You are not entirely aware of this)

**OPENING STATEMENT**:

**“I know that you all have ideas of what I am supposed to be doing, but I need to do what I**

**need to do.”**

**APPEARANCE AND BEHAVIOR**:

**Appearance**:

- Wearing street clothes and sitting at home in front of computer. Appears tired, bags under eyes. Occasional signs of lack of sleep (rubbing eyes or forehead, periodically yawning).

**Behavior:**

- You are tired and perhaps giving in to your situation.
- You are beginning to accept that you are not taking care of yourself, but this is the best you can do.
- You knowingly are not adhering to recommendations. Not because you do not want to comply, but because you find it too difficult.
- You want to do that right thing. Signing in to the telehealth visit is a demonstration that you want to take care of yourself, despite not being successful so far.

**Case Props**:

Small towel that can be rolled up as a lumbar support in chair.
See video as reference: <https://www.youtube.com/watch?v=LPDcl1RmQvE>

**HISTORY OF PRESENT ILLNESS**

You presented to the hospital ER just over 3 months ago because your back was hurting so badly that you could not drive your truck. You were diagnosed with a lumbar muscle strain, and treated with IV fluids and a muscle relaxant in the ER that immediately made you feel better. Your back is feeling better after rest and Ibuprofen. You started working with an interprofessional healthcare team, but work and your family’s needs got in the way. You missed several appointments. However, you know that your health is important, so you are doing another telehealth visit.

**YOUR UNDERSTANDING OF recent ER visit and Lumbar Muscle Strain**:

- You have bad posture.
- You should be careful when you are lifting things or helping people physically.
- You need to take medication to manage discomfort short term.
- You might get help from physical therapy and occupational therapy.
- You need to rethink your diet, exercise and self-care habits.
- You should be taking your other medicines.

**YOUR TOP HEALTH PRIORITIES that will slowly come out in conversation with team if asked:**

1. Controlling your back pain/maintaining no back pain so you can work and provide for family
2. Getting sleep – “I hate feeling tired”
3. Eating better – “I sometimes think I might feel less tired if I ate better”

**RESPONSE TO OTHER QUESTIONS**

If the student asks you if you are in a space that you feel comfortable speaking about their recent discharge from the hospital:

**“Yes, I am in my kitchen.”**

If the student asks you where your kids are:

**“My children are out.”**

If the student asks you where your father is:

**“My father is watching tv in another room.”**

If the student asks you to confirm your address:

**“Yes, it is the one that you have listed on the chart 124 Ridgefield in Rocky Point.”**

If the student asks you to confirm your phone number:

**“631-555-1234”**

If the student asks you if you understand the most recent recommendations:

**“Yes, our visit last time was very helpful.”**

If the student asks about your missed or rescheduled appointments:

**“I have just been so busy with work and I just have to put my dad and kids first.”**

If the student asks if they can explain anything again:

**“Sure, that’s fine. I’d appreciate a review.”**

If the student asks you about your diet:

**“I have been having BK and Wendy’s. I try to get the salads. But, I get the burgers too.”**

If the student asks you about any knowledge of posture and body mechanics:

**“I did put a towel roll in my truck like you said. I put it by my lower back, like in the handout you emailed.” (have towel roll in reach in case they ask to see how you rolled it up)**

If the student asks you about how the towel roll is working:

**“That actually works, unless I’m on a really long drive.”**

If the student asks what positions and exercises are best for your back:

**“I just know certain chairs bother me, so I am staying out of those.”**

If the student asks if you if you had your shoulder checked:

**“No. I haven’t had the time. ”**

If the student asks if you about your health priorities:

Concern about back pain:

**“When my back hurts I can’t do my job. I can’t take care of my dad and kids”**

Concern about Stress:

**“I don’t pay attention to stress. Do I have stress? I don’t know-maybe. What do you think?”**

Concern about Diet:

**“I try and make sure we’ve all got something to eat. Sometimes it’s good, sometimes it’s not so good”**

****Note:** At any time that it feels appropriate/natural, you may ask the student:

**“Why are you so concerned with what I know and don’t know? That’s not my problem. I just don’t have time”**

**PAST MEDICAL HISTORY**

**Medical Conditions**

High Blood Pressure

High Cholesterol

- You had been prescribed medication for your blood pressure that you are not taking consistently, any refills are done randomly. You want to manage the high cholesterol with diet changes, but it’s hard. You don’t like cooking. Maria did that stuff. You are thinking that maybe you need to start taking your medications more regularly, but you’re not sure what they are for.
- Prior to starting with the inter-professional group today, you have not seen a regular physician for more than 10 years.

Left Shoulder pain post slip and fall in shower, also recent pain post slip and fall in shower (you have not had time to get this checked out by a doctor)

Depression

**Mental Health**

You have been recommended to see a counselor for concern of depression in the past but have not yet seen one in person. You basically feel overwhelmed at caring for both your children and your father by yourself as well as having to be the sole provider for your family.

Additional information if asked:

- You usually sleep 5 hours a night, but at least once a week you have only been getting 4 hours total.
- You find joy in spending time with your children and friends at work
- You have not wanted to see a counselor for fear of social concerns of behavioral health and have been raised to “tough it out”
- You have fallen in shower “once or twice”
- You acknowledge you are depressed. You miss your wife but don’t necessarily connect this to depression.

**SUICIDAL IDEATION:** “I would never do that! My family needs me”

**PAST HOSPITALIZATIONS**

none.

**MEDICATIONS**

**Prescription Drugs:**

- Labetalol 25 mg daily - Taken in the AM (for High Blood Pressure diagnosed 10 years ago)
- Hydrochlorothiazide 25mg daily - Taken in the AM (for High Blood Pressure diagnosed 10 years ago)
- Lisinopril 10 mg daily - Taken in the AM (for High Blood pressure diagnosed 10 years ago)
- Wellbutrin for depression but did not fill prescription

**Over-the-Counter Drugs:**

- Ibuprofen – 200-600 mg for low back pain and occasional headaches and for pain you associate with day-to-day physical work and “getting older”.

**Illicit/Street Drugs:**

None. **“No, I would never do that!!”**

**SEXUAL HISTORY**

Not sexually active.

**LIFESTYLE/HABITS**

**Occupation:**

You are a food delivery driver. You consider yourself a reliable and good worker. You get along well with your coworkers and enjoy the social interaction. You have good health insurance through your work.

You enjoy your job and your colleagues but spend the majority of the day on the road in the truck.

You are concerned that the low back pain could affect your ability to drive your truck. The back pain is not new and you had been trying to intentionally plan driving to build in standing breaks. Standing makes you feel better.

**Alcohol:**

**“Yes”** You drink “a couple of beers every night”.

**Tobacco:**

**“Yes, I quit about 10 years ago, but started again after my wife (Maria) passed. It’s just been hard without her.”** If followup question: **“Only 2-3 a day.”**

**Caffeine:**

**“Yes”** You drink 1 cup of coffee per day in the morning. No tea, cola. Occasional chocolate bars.

**Diet:**

**“I love my sweets and have been eating them a long time. ”**

You don’t follow any special diet. You frequently snack on candy during the day or grab a quick meal on the road.

If asked to describe your typical eating for the day:

**Breakfast: “Something quick – I’ll pick up – usually a bagel or donut with a cup of coffee.”**

**Lunch: “Usually get something from the deli or the lunch truck – sandwich, soda, chips.”**

**Afternoon snack: “I like a candy bar midafternoon – I usually need a “pick me up**”

**Dinner: “Don’t cook much – most of the time I am too tired to eat after work.”** You are too tired after work and have to come home to take care of your family. Sometimes you will have a frozen meal, more often lately you will snack on chips or candy at home.

You have been told sometime in the past when your wife was alive to eat a balanced diet, less salt and watch the sugar. Maria helped with that, but since she passed it’s been hard and you have been working a lot to help provide for your family.

**Exercise:**

**“No”** You don’t have a regular exercise routine. You are too tired after work to exercise. You were also injured while exercising 6 years ago and still have difficulty raising your left arm over your head. If asked if the injury interferes with your job - you do not do a lot of exercise with your position at work.

**Activities/Hobbies:**

You like to read. No other hobbies as you are too tired after work.

**Sleep:**

- You get up early (6:00 am) to be at work by 8:00 am. You finish working by 5:00 pm and then come home to care for your father and frequently skip dinner. You are in bed early because of the early hour you get up.
- You are tired and not sleeping well. Your sleep has not been restful. You find yourself tired but having trouble falling asleep or you wake up in the middle of the night and have trouble falling back asleep. Average sleep at best 6 hours, but lately sometimes only 4-5 hours due to the trouble falling asleep or waking up. You sometimes find yourself nodding off after dinner for an hour.

**FAMILY MEDICAL HISTORY**

Your **father** is alive. He is 78 years old. He suffered a stroke 9 months ago and following five days of hospitalization he was discharged for three weeks to a rehabilitation center.  The rehabilitation center discharge plan, which included follow up medical and at-home physical and occupational therapy never happened. Your father requires help moving around the apartment and needs a walker to get around. While he can feed himself he does have limitations and is frequently forgetful to take his medications. You have been trained as an e-helper for his OT needs but your dad resists your care and is determined to do it on his own. Your dad has refused care from his grandchildren and you have not wanted to burden them by asking for help. The older one is in community college and works at night to help contribute and you value his education and don’t want to place too many demands on his time.

Your **mother** is deceased, died in a car accident 15 years ago. No brothers or sisters.

You did not have a close relationship with your **grandparents** because they lived in another city. Your **mother’s father died** of a heart attack at age 60. Your other grandparents are no longer alive and you are unaware of their health history. If asked, you did not get along with your wife’s side of the family, so have no information to offer clinicians with respect to care for your children.

**PERSONAL/FAMILY HISTORY**

- You are originally from Nicaragua and came to the U.S. with your family when you were 5.
- You grew up as an only child in a working lower economic class family in Queens.
- You have a high school education and have worked all your life as a truck driver.
- You met and married your deceased wife, Maria, in Queens and then moved to Rocky Point. You were married for 22 years until she died 5 years ago.
- You live with your three sons: 18, 16 and 12yo with whom you are very proud. They are in good physical health and all did well in school until your wife passed. You sense that all of them are “broken without their mother”. The children were 13, 11 and 7 when your wife died. The 18 year old successfully graduated high school, takes class at a community college and works at an auto body shop. When not working he is often with his girlfriend. You like the girlfriend. The 16 yo had been working at a pizzeria until recently has been skipping work and school. You were notified a couple of months ago that he could be in danger of repeating the 11^th^ grade. The 12 year old is very attached to you and your father. He is missing your oldest son.
- Your elderly father has lived with the family for about 15 years.
- You have lived in the same apartment for 30 years.
- The area is a socioeconomically disadvantaged urban neighborhood but you feel safe and have known your neighbors for years.
- You have struggled with food insecurity in the past. Your wife did the shopping and cooking. With her gone, you have relied more on fast food and candy snacks for yourself to be able to provide for your children. The family is doing a little better financially since your 18 and 16 year old started contributing financially, often taking it upon themselves to arrange their own meals.

**SCENARIO DEVELOPMENT**

**End of the encounter:**

If the inter-professional student teams says anything indicating that they have heard your concerns (i.e., they empathize) OR if they discuss ways, to address your concerns, you are agreeable to any suggestions.

You agree to a follow-up **telehealth** appointment, if that is suggested. **You agree to upload your any information or monitoring, if suggested.** If the inter-professional student team has done a good job of convincing you how important it is for you to follow recommendations you agree to do what is suggested. You would also agree to see other clinicians via telehealth if recommended.

**ADDITIONAL QUESTIONS YOU MAY ASK (if needed during the encounter) You do not need to ask all of these questions.**

1. If I stop driving, will my back pain go away?
2. Will I have it for the rest of my life?
3. How did I get it?
4. Is the back pain related to my shoulder?
5. What should I do to keep myself from getting worse?
6. Should I change my diet?
7. Should I start exercising?
8. If I exercise for my back and shoulder, will that help with my hypertension or my cholesterol?

**CHALLENGE QUESTION:**

If no one addresses or comments about how tired you appear you will ask:

**“Does sleep have anything to do with my health? ”**

If you are asked any questions that you aren’t aware of or if learners refer to any conversations they had with you in the past, you will respond **“I don’t remember talking about that. I have a lot going on.”**

IF any learner asks something that you do not know the answer to, you will respond: **“I’m really not sure” or “I don’t remember.”**

**TIMELINE**

- 10 years ago diagnosed with hypertension and high cholesterol
- 5 years ago your wife died
- 9 months ago your Father (who lives with you) suffered a stroke
- Just over 3 months ago you went to the ER with low back pain and were diagnosed with lumbar muscle strain. You were referred for an Interdisciplinary Telehealth Visit by community hospital ER. Identified as high risk for health problems and community needs
- Today you are having a Telehealth visit with the Interdisciplinary Telehealth Team after missing or rescheduling several appointments because you have been very busy.

**Scenario 3**

Pt. is 48 y.o. Hispanic male referred just over 3 months ago to interdisciplinary telehealth by the community hospital emergency department after evaluation for back pain as he was identified as “at risk” for multiple health problems and behavioral health needs for himself and his children. He had not been to an established clinic practice in years and had declined in person referrals to social work services, occupational therapy, physical therapy services and home care nursing. At the ER, Mr. Rivera was found to have lumbar muscle strain due to muscle strength and range of motion imbalance as well as poor body mechanics and postural deformities. He also experienced decreased range of motion from a previous injury to his left shoulder. He is aware he has high cholesterol and high blood pressure, both which are poorly controlled and acknowledges that he has mild to moderate depression.

His first visit focused on a team interview, baseline status and his back pain. The next visit prior to today focused on identifying his health priorities.

He is motivated to follow the advice of the health care team, but he feels that he does not have much time to take care of his needs. During his prior visit 2 weeks ago, he was tired and at a loss as to how he is supposed to fit health recommendations into his work and family life. He prioritizes helping his father with his father’s telehealth visits, over his own needs.
During the last visit, Frank identified that with respect to his health priorities he cares most about not having recurrent back pain, getting sleep and eating better.

Your challenges, as the standardized patient, include:

1. You will express that you need additional help to care for your father.
2. You will acknowledge that you are trying to prioritize yourself, but it is difficult. For example, finances are a barrier to filling your prescriptions.
3. You will share that you are having some trouble meeting the needs of parenting your school age sons. (“it seems like my 16 year old has been blowing off classes. I got a call from the principal about that”)
4. You will be clear that you do not want help taking care of your children’s needs (“the family can take care of the family” “this is no one else’s business” “I hope that you will not make me sorry that I am telling you this”
5. To observe the behaviors and communications of individual students through telehealth and the team as a whole while you are performing this case; and
6. To accurately recall the behaviors and communications and accurately complete the performance checklist.

The important challenges of this case for interprofessional student team are to:

1. Review the 1-3 of his health priorities from last week
2. Address ethical issues and prioritizing patient needs.
3. Assess Frank’s awareness of the social issues that he is facing
4. Assess Frank’s understanding of resources that may be available
5. Address 2 ethical dilemmas with Frank (identified by Frank or the team)
6. Provide collaborative care

To review:

Your main concerns are:

1) You need help taking care of your father
2) You need help finding a way to take care of yoursel
3) You do not want help taking care of your kids but recognize that they need support.

**OPENING STATEMENT**:

**“Well, I would be better if my father wasn’t so difficult. He has just really been something**

**lately… But I’m glad the internet’s working today. That’s a bonus.”**

**APPEARANCE AND BEHAVIOR**:

**Appearance**:

- Wearing street clothes and sitting at home in front of the computer.

**Behavior:**

- You are focused and interested in discussing how to get help for your father so you can take care of yourself
- You have no interest in advice about your kids, but you may vent about how they are having troubles.

**Case Props**:

None

**HISTORY OF PRESENT ILLNESS**

You presented to the hospital ER just over 3 months ago because your back was hurting so badly that you could not drive your truck. You were diagnosed with a lumbar muscle strain, and treated with IV fluids and a muscle relaxant in the ER that immediately made you feel better. Your back is feeling better after rest and Ibuprofen. You started working with an interprofessional healthcare team, but work and your family's needs got in the way. You missed several appointments. However, you know that your health is important so you are doing another telehealth visit.

**YOUR UNDERSTANDING OF lumbar muscle strain and your general health**:

- You have bad posture and should be careful when you are lifting things or helping people physically.
- You need to rethink your diet, exercise and self-care habits.
- You should be taking your other medicines.

**YOUR TOP HEALTH PRIORITIES shared in some form from last visit:**

1. Controlling your back pain/no back pain so you can work and provide for family
2. Getting sleep
3. Eating better

**RESPONSE TO OTHER QUESTIONS**

If the student asks you if you are in a space that you feel comfortable speaking about their recent discharge from the hospital:

**“Yes, I am in my kitchen.”**

If the student asks you where your kids are:

**“My children are out.”**

If the student asks you where your father is:

**“My father is watching tv in another room.”**

If the student asks you to confirm your address:

**“Yes, it is the one that you have listed on the chart 124 Ridgefield in Rocky Point.”**

If the student asks you to confirm your phone number:

**“631-555-1234.”**

If the student asks you if you recall the most recent visit:

**“I know that we talked about how I am just a mess, you know my back, my diet and my crummy sleep.”**

If the student asks you if you recall the information from last visit**:**

**“I know you think I’m stressed. I probably am. I wish I could spend time working on these things but I just need to take care of my dad.”**

If the student asks about your missed or rescheduled appointments:

**“I have just been so busy with work and I just have to put my dad and kids first.”**

If the student asks if they can explain anything again:

**“Sure, that’s fine. I’d appreciate a review.”**

If the student asks about your sleeping:

**“I have been working on that and I have been sleeping better. I may not exercise but I am working on that sleep.”**

If the student asks how you have been working on your sleeping:

**“I have been avoiding naps and trying to wake up at the same time every day.”**

If the student persists with questions about sleeping:

**“My son told me he saw something about that being good, something on social media.”**

If the student asks you about your diet:

**“I have been only doing salad when I do fast food, or I get grilled chicken.”**

If the student asks you about frequency of cooking:

**“I cooked twice last week. My son’s girlfriend cooked for us once also.”**

If the student asks you about how he thinks about home cooking versus takeout:

**“When his girlfriend makes tacos, it is good. She makes it with chicken though. She said that was healthy. What do you think? I don’t know. It tasted good.”**

If the student asks you about any knowledge of posture and body mechanics:

**“I use my towel roll. I am careful about picking up heavy stuff.”**

If the student asks what positions and exercises are best for your back:

**“Not working (kidding). Standing and walking are the best. Not a lot of sitting. Using the lumbar roll.”**

If the student asks if you if you had your shoulder checked:

**“Nope. Still haven’t done that.”**

If the student asks you about your ability to afford your medications:

**“Well I have told you I can’t afford my meds. I know you say it’s important but we need food and my dad has medications too. I need to provide for them.”**

If the student asks you about how you care for your dad during the day:

**“Well, he stays in the house pretty much. If I’m working my dad is mostly in his room.”**

If the student asks you about how you feel about help for your dad:

**“He really is kind of set in his ways. You know he wants things his way. He pretty much refuses to let anyone else come in to help him besides me. Even the kids - he doesn’t like them to help.”**

If the student asks you about things that you have tried or considered in the past, or resources related to your dad:

**“I have tried to have people come from the church, people from the neighborhood that he knows.”**

If the student asks you how often the telehealth visits are for your dad:

**“The rehab sessions are once a week, however I am only able to make the sessions now once every 3 weeks due to my Dad’s resistance to the visit.”**

If the student asks you about what you think your dad could use help with:

**“Like he stinks and needs a bath and he doesn’t want to wash up. I have to really convince him to take a bath.”**

If the student asks you about your kids and their well-being/welfare:

You will initially be abrupt/defensive/borderline offended:

**“I’m doing my best. Could they be better? Probably, had their mother not died. But it is what it is.”**

If students continue to probe you can start to open up and share your worries:

“**Look I’m honestly really worried about my two youngest.”**

If students ask you what you are worried about:

“**My 16 year old is skipping school. I got this note the other day, a really serious letter, that if he doesn’t show up, he’s going to have to repeat 11th grade.”**

If students ask you again what you are worried about or probe more:

**“I leave before they get up in the morning. It’s dinner or later when I get home. I hope that you’re not going to report me now that I shared this with you.”**

If students ask you why they would report you:

**“I’ve heard how that goes when families get listed in the system. I don’t want to lose my kids.”**

If the student asks you about the safety of your apartment:

**“I know what you mean. This place isn’t the best in our situation. But, I don’t want to disrupt our lives any more than it already has been since my wife died. This is our home.”**

If the student asks you about the priority of your health versus your father:

**“Well, he’s my responsibility. He’s my father.”**

If the student asks you about what would happen to your dad if something happened to you:

**“Well, I don’t know. I actually try NOT to think about that. He probably would be put in a home.”**

****Note:** At any time that it feels appropriate/natural, you may ask the student:

**“I just don’t know what to do anymore. I’m feeling really overwhelmed.”**

**PAST MEDICAL HISTORY**

**Medical Conditions**

High Blood Pressure

High Cholesterol

- You have been prescribed medication for your blood pressure that you are not taking consistently, any refills are done randomly. You want to manage the high cholesterol with diet changes, but it’s hard. You don’t like cooking. Maria did that stuff. You are thinking that maybe you need to start taking your medications more regularly, but you’re not sure what they are for.
- Prior to starting with the inter-professional group today, you have not seen a regular physician for more than 10 years.

Left Shoulder pain post slip and fall in shower (you have not had time to get this checked out by a doctor)

Depression

**Mental Health**

You have been recommended to see a counselor for concern of depression in the past but have not yet seen one in person. You basically feel overwhelmed at caring for both your children and your father by yourself as well as having to be the sole provider for your family.

Additional information if asked:

- You sleep 6-7 hours a night now. You have been working on this.
- You find joy in spending time with your children and friends at work
- You have not wanted to see a counselor for fear of social concerns of behavioral health and have been raised to “tough it out”
- You have fallen in shower “once or twice”

**SUICIDAL IDEATION:** “I would never do that! My family needs me”

**PAST HOSPITALIZATIONS**

none.

**MEDICATIONS**

**Prescription Drugs:**

- Labetalol 25 mg daily - Taken in the AM (for High Blood Pressure diagnosed 10 years ago)
- Hydrochlorothiazide 25mg daily - Taken in the AM (for High Blood Pressure diagnosed 10 years ago)
- Lisinopril 10 mg daily - Taken in the AM (for High Blood pressure diagnosed 10 years ago)
- Wellbutrin for depression but did not fill prescription

**Over-the-Counter Drugs:**

- Ibuprofen -200-600 mg for low back pain and occasional headaches and for pain you associate with day-to-day physical work and “getting older”. Have not been taking this recently.

**Illicit/Street Drugs:**

None. **“No, I would never do that!!”**

**SEXUAL HISTORY**

Not sexually active.

**LIFESTYLE/HABITS**

**Occupation:**

You are a food delivery driver. You consider yourself a reliable and good worker. You get along well with your coworkers and enjoy the social interaction. You have good health insurance through your work.

You enjoy your job and your colleagues but spend the majority of the day on the road in the truck.

You are concerned that the low back pain could affect your ability to drive your truck. The back pain is not new and you had been trying to intentionally plan driving to build in standing breaks. Standing makes you feel better.

You work four12 hour shifts a week. You are sometimes able to pick up one or two overtime shifts a month however with your back hurting you have not been able to do that recently.

**Alcohol:**

**“Yes”** You drink “a couple of beers every night”.

**Tobacco:**

**“Yes, I quit about 10 years ago, but started again after my wife (Maria) passed. It’s just been hard without her.”** If follow-up question: **“Only 2-3 a day.”**

**Caffeine:**

**“Yes” You drink 1 cup of coffee per day in the morning. No tea, cola. Occasional chocolate bars.**

**Diet:**

**“I love my sweets and have been eating them a long time.”** You don’t follow any special diet. You frequently snack on candy during the day or grab a quick meal on the road.

If asked to describe your typical eating for the day:

**Breakfast**: “Something quick – I’ll pick up – usually a bagel or donut with a cup of coffee.”

**Lunch**: “Usually get something from the deli or the lunch truck – sandwich, soda, chips.”

**Afternoon snack:** “I like a candy bar midafternoon – I usually need a “pick me up”

**Dinner**: “Don’t cook much – most of the time ~~you are~~ I am too tired to eat after work.” You are too tired after work and have to come home to take care of your family. Sometimes you will have a frozen meal, more often lately you will snack on chips or candy at home.

You have been told sometime in the past when your wife was alive to eat a balanced diet, less salt and watch the sugar. Maria helped with that, but since she passed it’s been hard and you have been working a lot to help provide for your family.

**Exercise:**

**“No”** You don’t have a regular exercise routine. You are too tired after work to exercise. You were also injured while exercising 6 years ago and still have difficulty raising your left arm over your head. If asked if the injury interferes with your job - you do not do a lot of exercise with your position at work.

**Activities/Hobbies:**

You like to read. No other hobbies as you are too tired after work.

**Sleep:**

- You get up early (6:00 or 7:00 am) to be at work by 8:00 am. You finish working by 5:00 pm and then come home to care for your father and frequently skip dinner. You are in bed early because of the early hour you get up.
- Your sleep has been better since you have less back pain. You are getting 6-7 hours of sleep. Your sleep has been more restful. And you have been avoiding naps, and consistently waking up at 6am.

**FAMILY MEDICAL HISTORY**

Your **father** is alive. He is 78 years old. He suffered a stroke 9 months ago and following five days of hospitalization he was discharged for three weeks to a rehabilitation center.  The rehabilitation center discharge plan, which included follow up medical and at-home physical and occupational therapy never happened. Your father requires help moving around the apartment and needs a walker to get around. While he can feed himself, he does have limitations and is frequently forgetful to take his medications. You have been trained as an e-helper for his OT needs but your dad resists your care and is determined to do it on his own. Your dad has refused care from his grandchildren and you have not wanted to burden them by asking for help. The older one is in community college and works at night to help contribute and you value his education and don’t want to place too many demands on his time.

Your **mother** is deceased, died in a car accident 15 years ago. No brothers or sisters.

You did not have a close relationship with your **grandparents** because they lived in another city. Your **mother’s father died** of a heart attack at age 60. Your other grandparents are no longer alive and you are unaware of their health history. If asked, you did not get along with your wife’s side of the family, so have no information to offer clinicians with respect to care for your children.

**PERSONAL/FAMILY HISTORY**

- You are originally from Nicaragua and came to the U.S. with your family when you were 5.
- You grew up as an only child in a working lower economic class family in Queens.
- You have a high school education and have worked all your life as a truck driver.
- You met and married your deceased wife, Maria, in Queens and then moved to Rocky Point. You were married for 22 years until she died 5 years ago.
- You live with your three sons: 18, 16 and 12yo with whom you are very proud. They are in good physical health and all did well in school until your wife passed. You sense that all of them are “broken without their mother”. The children were 13, 11 and 7 when your wife died. The 18 year old successfully graduated high school, takes class at a community college and works at an auto body shop. When not working he is often with his girlfriend. You like the girlfriend. The 16 yo had been working at a pizzeria until recently has been skipping work and school. You were notified a couple of months ago that he could be in danger of repeating the 11^th^ grade. The 12 year old is very attached to you and your father. He is missing your oldest son.
- Your elderly father has lived with the family for about 15 years.
- You have lived in the same apartment for 30 years.
- The area is a socioeconomically disadvantaged urban neighborhood but you feel safe and have known your neighbors for years.
- You have struggled with food insecurity in the past. Your wife did the shopping and cooking. With her gone, you have relied more on fast food and candy snacks for yourself to be able to provide for your children. The family is doing a little better financially since your 18 and 16 year old started contributing financially, often taking it upon themselves to arrange their own meals.

**SCENARIO DEVELOPMENT**

**End of the encounter:**

If the inter-professional student teams says anything indicating that they have heard your concerns (i.e., they empathize) OR if they discuss ways to address your concerns, you are agreeable to any suggestions.

You agree to a follow-up **telehealth** appointment, if that is suggested. **You agree to upload your any information or monitoring, if suggested.** If the inter-professional student team has done a good job of convincing you how important it is for you to follow recommendations you agree to do what is suggested. You would also agree to see other clinicians via telehealth if recommended.

**ADDITIONAL QUESTIONS YOU MAY ASK ( if needed during the encounter) You do not need to ask all of these questions.**

1. What do other people do when their parents get old and angry?
2. How would I get care for my father, if something happened to me?

**CHALLENGE QUESTION:**

If no one addresses or comments about how you might get help for your children you will ask:

**“If I wanted it, and I’m not saying I want it, is there any help that I could get with taking care of my kids. And what would that look like?”**

IF any learner asks something that you do not know the answer to, or a prior conversation, you may respond: **“I’m really not sure” or “I don’t remember”**

**TIMELINE**

- 10 years ago, diagnosed with hypertension and high cholesterol
- 5 years ago, your wife died
- 9 months ago, your Father (who lives with you) suffered a stroke
- Just over 3 months ago you went to the ER with low back pain and were diagnosed with lumbar muscle strain. You were referred for an Interdisciplinary Telehealth Visit by community hospital ER. Identified as high risk for health problems and community needs
- 2 weeks ago, you had a Telehealth visit with the Interdisciplinary Telehealth Team after missing or rescheduling several appointments because you have been very busy.
- Today (2 weeks later) you agreed to have another Telehealth visit with the Interdisciplinary Team to follow up.

**Scenario 4**

Frank Rivera is a 48-year-old Hispanic male who was discharged two days ago from the hospital after being admitted because of symptoms of dizziness. He was diagnosed with type 2 diabetes. He is widowed from his wife, Maria, and lives with his three children and his elderly father. Mr. Rivera is being discharged on Metformin 500mg twice a day, this is a new medication for him. He has also recently learned how to check his blood sugar and use a glucometer during this hospitalization. Until this hospital admission, he has never taken any medications for diabetes so having to check his blood sugar is new for him. He is not confident in how to use the glucometer and still has not yet picked it up from the pharmacy.

Frank is motivated to follow the advice of the health care team, but he is feeling overwhelmed about the disease process and management. Despite their education previously, he has not been following a healthy diet (eats a lot of carbohydrates and concentrated sweets). He has also not been exercising, since he finds it hard to set aside time for it. He is worried that he will forget to take his medications properly and that he will “mess up” when checking his blood sugar. He has relatives with diabetes and has seen that complications arise if blood sugar is not properly controlled.

His first visit was 4 months ago with this team and focused on a team interview, baseline status and his back pain. The 2^nd^ visit focused on identifying his health priorities this was 4 weeks ago. The 3^rd^ visit was 2.5 weeks ago that focused on reviewing his health priorities, and discussing ethical issues to help prioritize Frank’s health needs above that of his father and children.

The main goal of this interprofessional student team is to ensure a safe discharge process utilizing telehealth for Mr. Rivera. The healthcare student team is instructed to determine the patient's understanding of his diagnosis of diabetes, his home management including checking and logging his blood sugar, and his knowledge and management of complicating factors such as social support network, and access to resources.

Your challenges, as the standardized patient, include:

1. To demonstrate uncertainty with the overall plan: when to check blood sugar, how often to record blood sugar readings, and why it is important to change your diet, and continue not to smoke.
2. You are also unclear about what the new medication (Metformin) will do – you have been taught to watch for signs of low blood sugar – but you are unsure if you understand what the signs mean and what to do if you develop them
3. To observe the behaviors and communications of individual students through telehealth and the team as a whole while you are performing this case; and
4. To accurately recall the behaviors and communications and accurately complete the performance checklist.

The important challenges of this case for inter-professional student team are to:

1. Explain the diagnosis, treatment, and importance of treatment to the patient in plain language.
2. Assess the patient's understanding of his diagnosis (what is the disease)
3. Assess the patient’s understanding of the treatment (role of the medication, how to take it, possible side effects)
4. Assess the patient’s understanding of self-care and nutrition (how and when to check blood sugar, dietary changes to be made, dental evaluation, checking feet, quit smoking, exercising and making follow-up appointments)
5. Assess the patient’s understanding of diabetes and why taking the medication and checking the blood sugar, maintaining a health diet and exercise are important
6. Provide collaborative care in conducting a safe patient discharge.
7. Ensure comfortability with using telehealth technology for follow-up

To review:

Your main concerns are:

1) You are overwhelmed with all the new material you’ve been given
2) You are resistant to any dietary changes because of this one episode of dizziness
3) You do not know how you will add checking your blood sugar into your routine while at work as you are on a tight schedule
4) You are concerned about when and how to take your new medication
5) You are concerned about how potential vision changes and foot problems may affect your work.

The reasons for your dietary and exercise indiscretion include:

1. You love sweets (donuts in the AM, candy bars with every lunch)

2. You’re not sure exactly what a diabetic diet is

3. You’re not sure what difference a change in diet would make as you feel unwell based on this one episode of dizziness

4. You have time constraints, often tired and not properly prepared (shopping, meal and snack planning, exercise planning)

**OPENING STATEMENT**:

**“They gave me this discharge plan, but to tell you the truth I’m feeling a little**

**overwhelmed.”**

**APPEARANCE AND BEHAVIOR**:

**Appearance**:

- Wearing street clothes and sitting at home in front of computer

**Behavior:**

- You are overwhelmed with the amount of information you have been given regarding your diagnosis and treatment plan for discharge
- You appear confused and slightly anxious but cooperative during the encounter
- You also are slightly tired from the transition back to home after the hospital and have not been sleeping well, only about 5 hours a night.
- You are feeling stressed because you are the primary caregiver for the family including your elderly father.

**Case Props**:

You will have a copy of your Individualized Patient Discharge Plan (found on the last page of the case).

**HISTORY OF PRESENT ILLNESS**

You were admitted to the hospital two days ago after presenting to the emergency department complaining of an acute onset of dizziness and nausea. You were concerned because you have a history of high blood pressure and were concerned about a heart attack. You also noticed you’ve been urinating more frequently and have been more thirsty than usual. You drove yourself to the hospital despite your symptoms (you do not like to call 911 or bother people unless it is absolutely necessary). You were discharged from the hospital now that the medical team has gotten your blood sugar controlled.

During the hospitalization, you were diagnosed as having diabetes but have limited understanding of what diabetes does to your body and how to manage it. Prior to the last 4 months you had barely seen a physician. Over the last four months you have been trying out telehealth, seeing members of the inter-professional healthcare team on a computer. You have had 3 visits with the team so far and the last visit was 2.5 weeks ago.

**YOUR UNDERSTANDING OF DIABETES**:

- You have too much sugar in your blood
- You need to take medication to control it
- You need to use the machine to monitor your blood sugar
- You need to change your diet and should stay away from sweets

**RESPONSE TO OTHER QUESTIONS**

If the student asks you if you are in a space that you feel comfortable speaking about their recent discharge from the hospital:

**“Yes I am in my living room.”**

If the student asks you where your kids are:

**“My children are out”**

If the student asks you where your father is:

**“My father is watching tv in another room.”**

If the student asks you to confirm your address:

**“Yes, it is the one that you have listed on the chart 124 Ridgefield in Rocky Point.”**

If the student asks you if you have your glucometer for home:

**“No I have to pick it up from the pharmacy”**

If the student asks you to confirm your phone number:

**“631-555-1234”**

If the student asks you if you understand your discharge instructions:

**“Not totally. They told me so many things that it is a little overwhelming.”**

If the student asks if they can explain anything again:

**“Yes please! I’m feeling so overwhelmed with information right now. I’m not even sure when or how they said I should check my sugar.”**

If the student asks if you know how to use the glucometer:

**“They showed me how to use it but I’m not sure how often. I have not even had a chance to pick it up from the pharmacy”**

If the student asks you about your diet:

**“I have been only doing salad when I do fast food, or I get grilled chicken.”**

If the student asks you more about your diet:

**“I love my sweets and been eating them a long time.**

If the student asks you about any knowledge of diabetic diets:

**“Oh I know I’m supposed to stay away from the sweets. I know I’m bad for eating all that junk, but I’ve been eating them all my life.**

If the student asks what your blood sugar should be:

**“I think around 100.”**

If the student asks if you know the signs of hypoglycemia or low blood sugar, you will say:

**“I think they said I’ll feel weak.”**

****Note:** At any time that it feels appropriate/natural, you may ask the student:

**“Why are you so concerned about my diet?** **Honestly, I feel pretty good now and I feel better when I eat candy.”**

**PAST MEDICAL HISTORY**

**Medical Conditions**

High Blood Pressure

High Cholesterol

- You had been prescribed medication for your blood pressure that you were not taking consistently, any refills were done randomly. You wanted to manage the high cholesterol with diet changes. You are starting to take your medications more regularly the last 4 months since starting with the inter-professional group.
- Prior to starting with the inter-professional group 4 months ago, you had not seen a regular physician for more than 10 years.

Lumbar muscle strain w/low back pain (evaluated in ER 4 months ago)

Left Shoulder pain post slip and fall in shower (you have not had time to get this checked out by a doctor)

Depression

**Mental Health**

You have been recommended to see a counselor for concern of depression in the past but have not yet seen one in person. You basically feel overwhelmed at caring for both your children and your father by yourself as well as having to be the sole provider for your family.

Additional information if asked:

- You sleep 5 hours a night
- You find joy in spending time with your children and friends at work
- You have not wanted to see a counselor for fear of social concerns of behavioral health and have been raised to “tough it out”
- You have fallen in shower “once or twice”)

**PAST HOSPITALIZATIONS**

ER visit 4 months ago for back pain. Otherwise none.

**MEDICATIONS**

**Prescription Drugs:**

- Labetalol 25 mg daily - Taken in the AM (for High Blood Pressure diagnosed 10 years ago)
- Hydrochlorothiazide 25mg daily - Taken in the AM (for High Blood Pressure diagnosed 10 years ago)
- Lisinopril 10 mg daily - Taken in the AM (for High Blood pressure diagnosed 10 years ago)
- Wellbutrin for depression but did not fill prescription
- Metformin 500mg twice a day

**Over-the-Counter Drugs:**

- Ibuprofen – 200-600 mg for low back pain and occasional headaches and for pain you associate with day-to-day physical work and “getting older”. Have not been taking consistently

**Illicit/Street Drugs:**

None. **“No, I would never do that!!”**

**SEXUAL HISTORY**

Not sexually active.

**LIFESTYLE/HABITS**

**Occupation:**

You are a food delivery driver. You consider yourself a reliable and good worker. You get along well with your coworkers and enjoy the social interaction. You have good health insurance through your work.

You enjoy your job and your colleagues but spend the majority of the day on the road in the truck.

You are concerned if the diabetes will affect your vision as you need your CDL license to drive a truck.

You work four10 hour shifts a week. You are sometimes able to pick up one or two overtime shifts a month however with your back hurting you have not been able to do that recently.

**Alcohol:**

**“Yes”** You drink “a couple of beers every night”.

**Tobacco:**

**“Yes, I quit about 10 years ago, but started again after my wife passed. She wouldn’t like that I am smoking again. It’s just been hard without her.”** If follow-up question: **“Only 2-3 a day.”**

**Caffeine:**

**“Yes”** You drink 1 cup of coffee per day in the morning. No tea, cola. Occasional chocolate

bars.

**Diet:**

**“I love my sweets and have been eating them a long time. Can you explain to me how in the world they expect me to start eating different?”**

You don’t follow any special diet, and you eat sweets frequently. You frequently snack on candy during the day or grab a quick meal on the road.

If asked to describe your typical eating for the day:

**Breakfast**: “Something quick – I’ll pick up – usually a bagel or donut with a cup of coffee.”

**Lunch**: “Usually get something from the deli or the lunch truck – sandwich, soda, chips.”

**Afternoon snack:** “I like a candy bar midafternoon – I usually need a “pick me up”

**Dinner**: “Don’t cook much – most of the time ~~you are~~ I am too tired to eat after work.” You are too tired after work and have to come home to take care of your family. Sometimes you will have a frozen meal, more often lately you will snack on chips or candy at home.

You have been counseled recently about improving food choices and eating a more balanced diet but change has been hard and you have been working a lot to help provide for your family.

**Exercise:**

**“No”** You don’t have a regular exercise routine. You are too tired after work to exercise. You were also injured while exercising 6 years ago and still have difficulty raising your left arm over your head. If asked if the injury interferes with your job - you do not do a lot of exercise with your position at work.

**Activities/Hobbies:**

You like to read. No other hobbies as you are too tired after work.

**Sleep:**

- You get up early (6:00 am) to be at work by 7:00 am. You finish working by 5:00 pm and then come home to care for your father and frequently skip dinner. You are in bed early because of the early hour you get up.
- You also are slightly tired from the transition back to home after the hospital and have not been sleeping well, only about 5 hours a night.

**FAMILY MEDICAL HISTORY**

Your **father** is alive. He is 78 years old. He suffered a stroke twelve months ago and following five days of hospitalization he was discharged for three weeks to a rehabilitation center.  The rehabilitation center discharge plan, which included follow up medical and at-home physical and occupational therapy never happened. Your father requires help moving around the apartment and needs a walker to get around. While he can feed himself he does have limitations and is frequently forgetful to take his medications. You have been trained as an e-helper for his OT needs but your dad resists your care and is determined to do it on his own. Your dad has refused care from his grandchildren and you have not wanted to burden them by asking for help. The older two are in community college and work at night to help contribute and you value their education and don’t want to place too many demands on their time. You have been working with the inter-professional team on getting resources for your father and prioritizing your own care. You are now worried to add one more thing to your growing list of responsibilities at home like checking your blood sugar.

Your **mother** is deceased, died in a car accident 15 years ago. No brothers or sisters.

You did not have a close relationship with your **grandparents** because they lived in another city. Your **mother’s father died** of a heart attack at age 60. Your other grandparents are no longer alive and you are unaware of their health history. You did not get along with your wife’s side of the family, so have no information to offer clinicians with respect to care for your children.

**PERSONAL/FAMILY HISTORY**

- You are originally from Nicaragua and came to the U.S. with your family when you were 5.
- You grew up as an only child in a working lower economic class family in Queens.
- You have a high school education and have worked all your life as a truck driver.
- You met and married your now deceased wife, Maria, in Queens and then moved to Rocky Point. You were married for 22 years until she died 5 years ago.
- You live with your three sons: 18, 16 and 12yo with whom you are very proud. They are in good physical health and all did well in school until your wife passed. You sense that all of them are “broken without their mother”. The children were 13, 11 and 7 when your wife died. The 18 year old successfully graduated high school and works at an auto body shop. When not working he is often with his girlfriend. You like the girlfriend. The 16 yo had been working at a pizzeria until recently has been skipping work and school. You were notified a couple of months ago that he could be in danger of repeating the 11^th^ grade. The 12 year old is very attached to you and your father. He is missing your oldest son.
- Your elderly father has lived with the family for about 15 years.
- You have lived in the same apartment for 30 years.
- The area is a socioeconomically disadvantaged urban neighborhood but you feel safe and have known your neighbors for years.
- You have struggled with food insecurity in the past. Your wife did the shopping and cooking. With her gone, you have relied more on fast food and candy snacks for yourself to be able to provide for your children. The family is doing a little better financially since the your 18 and 16 year old started contributing financially, often taking it upon themselves to arrange their own meals.

**SCENARIO DEVELOPMENT**

**End of the encounter:**

If the inter-professional student teams says anything indicating that they have heard your concerns regarding the difficulty of checking your blood sugar regularly (i.e., they empathize) OR if they discuss ways, other than drugs, to control your blood sugar (weight control, improving your oral health to assist with a change in diet, exercise), you are agreeable to any suggestions.

You agree to a follow-up **telehealth** appointment, if that is suggested. **You agree to upload your sugar readings to the patient portal for close sugar monitoring, if suggested.** If the inter-professional student team has done a good job of convincing you how important it is for you to check your blood sugar regularly and to adhere to a diabetic diet you agree to see a nutritionist/diabetic educator. You would also agree to see a podiatrist if recommended.

**ADDITIONAL DIABETES QUESTIONS YOU MAY ASK ( if needed during the encounter) You do not need to ask all of these questions.**

1. If I stop eating candy and take this medicines, will my diabetes go away?
2. Will I have it for the rest of my life?
3. How did I get it?
4. What are other signs of diabetes I should look out for?
5. What are the chances I can go blind or have a leg amputated? I heard a friend say that’s what happened to his sister.
6. What is an “A1C” test?
7. What does this test tell you about how I am doing?
8. What should I do to keep myself from getting worse?
9. Should I change my diet?
10. Should I start exercising?
11. Can I give it to my children?
12. What are the signs of high blood sugar?
13. What are the “other” signs for low blood sugar?
14. What are the side effects of taking diabetes pills?

**CHALLENGE QUESTION:**

If no one addresses the signs and/or treatment of hypoglycemia you will ask:

**“So what am I supposed to do if my sugar gets too low?”**

If you are asked any questions you aren’t aware of or if learners refer to any conversations they had with you in the past, you will respond **“I don’t remember talking about that. I guess I’m just overwhelmed, I have a lot going on.”**

**TIMELINE**

- 10 years ago diagnosed with hypertension and high cholesterol
- 5 years ago your wife died
- 1 year ago your Father (who lives with you) suffered a stroke
- 4 months ago you went to the ER with low back pain and were diagnosed with lumbar muscle strain. You were referred for an Interdisciplinary Telehealth Visit by community hospital ER. Identified as high risk for health problems and community needs
- 1 month ago you had your 2^nd^ meeting with the Interdisciplinary Telehealth Team. You reported feeling overwhelmed with the care of your Father, not really addressing your health needs
- 2.5 weeks ago you had your 3^rd^ meeting with the Interdisciplinary Telehealth Team. At this meeting you reported that you were attempting to prioritize some of your self-care needs.
- 4 days ago you went to the ER with sudden onset of dizziness and nausea. You were diagnosed with diabetes
- 2 days ago you were discharged home from hospital
- Today you are having a Telehealth visit with the Interdisciplinary Telehealth Team to ensure your transition back home is going well.
