# Supplementary material for: Virtual Interprofessional Telehealth Education: Mixed Methods Evaluation
Source: JMIR Form Res. 2026 Aug 13;10:e92077. doi: 10.2196/92077 (PMC13472524; doi:10.2196/92077)
Supplement: Supplementary file 4 [file formative-v10-e92077-s004.docx]

**Door Instructions**

**Scenario 1:**

Pt. is 48 y.o. Hispanic male originally from Nicaragua being referred to interdisciplinary telehealth visit. Referred by the community hospital emergency department after evaluation for back pain as he was identified as “at risk” for multiple health problems and behavioral health needs for himself and his children. He has not been to an established clinic practice in years and has declined in-person referrals to social work services, occupational therapy, physical therapy services and home care nursing. Pt. was found to have lumbar muscle strain due to muscle strength and range of motion imbalance as well as poor body mechanics and postural deformities. Frank also experiences decreased range of motion from a previous injury to his left shoulder he had six years ago while exercising. He describes maybe being overeager and aggressively used barbells that were too heavy for too many reps. He did not seek medical care because he did not have time or money. He endorses that more recently he slipped and fallen in the shower “once or maybe twice” which may or may not have added to the left arm injury. He is aware he has high cholesterol and high blood pressure, both which are poorly controlled and acknowledges that he has mild to moderate depression. He is supposed to be on ibuprofen (anti-inflammatory), labetalol (Beta blocker), Hydrochlorothiazide (Diuretic), lisinopril (ACE Inhibitor). It was recommended to Frank to start Wellbutrin for depression however he did not fill the prescription. In addition, Frank has been advised to quit smoking on multiple occasions. Frank lives with his three children (18,16, 12) in a small apartment with a medically fragile father who has had a stroke in the last year. Frank’s wife passed away 5 years ago. Frank drives a food delivery truck for a living and can only afford an apartment in a resource poor and low socioeconomic area for his family. Prior documentation reveals that Frank often indicates that he is quite proud of his Latinx heritage and offers a rationale that he does not seek care because “the family takes care of the family above all else”.

**Scenario 2:**

It has been 3 months since the entire IPE team has met with Frank. He has missed several telehealth appointments for a variety of reasons related to the multiple demands on his time. Team members agree that when they happen to meet with Frank, he seems more focused on his father’s health care than his parenting responsibilities and his own care. Frank reports feeling overwhelmed and stuck when faced with his duties as an e-helper for his father's telehealth rehabilitation sessions (“they ask him to do so much stuff which winds up landing on me”). Frank acknowledges that he probably pays too much attention to his father’s needs and not enough to his own or his children’s but brushes it off by affirming that he is doing what a “good son” would do. He briefly shares that he has never felt like he can do enough for his family.

Team members also report Frank’s limited progress in his own care plans.

**Scenario 3:**

Frank states he has been attempting to prioritize some of his self-determined self-care needs however it remains a challenge in balancing with the needs of his father. He reluctantly reports he had not filled all his medications due to cost. Frank believes his father is declining in function and his ability to remember the simplest things. In addition, his father is on occasion refusing to attend telehealth rehabilitation sessions and is experiencing more frequent episodes of anger and resistance to daily care including feeding and bathing. The school district has contacted Frank to inform him that his 16-year son has been skipping classes and is in danger of repeating 11^th^ grade. He also has not followed up on the referral for evaluation of his 12-year old’s learning and behavioral health needs as reported by the school. He asks the team not to “report him” asking that you respect his belief (“the family can take care of the family”) over the systems and policies that surround his children’s education.

Frank recognizes he needs additional help in caring for his father, but his father refuses to let anyone but Frank take care of him. Attending the telehealth sessions for his father is a large constraint on his time and has intermittently been a financial burden as Frank does not have all the minutes in his plan to allow for his family to have the daily use, as well as, need the extra time for visits. His father does not accept the assistance of his grandchildren when they offer and refuses to let a family friend and neighbor in his room. Frank would like to keep his dad at home, but he is finding this increasingly challenging. The apartment building he is in has not had a working elevator for a year and he does not know how to approach other needed safety renovations to his unit. Frank’s internet has become unstable due to a broken router on the roof. The government insurance that Frank has enrolled his dad in would cover the costs of a skilled nursing facility, however Frank is conflicted about this decision and his dad will not hear of it. On questioning, his dad does not have a healthcare proxy and does not see the need for one, and furthermore he does not understand the concept of providers on a computer, and it is challenging to build a trusting patient-provider relationship.

**Scenario 4:**

Frank Rivera, a 48-year-old male, was discharged from the hospital two days ago after a two-day admission because of complaints of dizziness. During his hospital stay Frank was diagnosed with type 2 diabetes and was discharged on Metformin 500mg twice a day. This is a new medication for him. In the hospital, Frank learned how to check his blood sugar and use a glucometer. You are members on a general medicine telehealthcare team, that have been following him in the community for 4 months, and have a scheduled telehealth visit to ensure his transition back home is going well. A copy of the discharge instructions is provided for you. Your overall task is to ensure that he is good at home—that he understands his diagnosis, the treatment plan, and the importance of adhering to his treatment plan.
