# Supplementary material for: Virtual Interprofessional Telehealth Education: Mixed Methods Evaluation
Source: JMIR Form Res. 2026 Aug 13;10:e92077. doi: 10.2196/92077 (PMC13472524; doi:10.2196/92077)
Supplement: Supplementary file 5 [file formative-v10-e92077-s005.docx]

**Timeline for Interprofessional Student Team**

| Stage | Time Allotted | Action/Tasks | Team Group Interviewing the Patient | Team Group Observing |
| --- | --- | --- | --- | --- |
| 1 | 15 min: | Team Planning Huddle  Review Tuesdays Plan (challenge: they do not know which team will interview patient first) | Groups A and B |  |
| 2 | 20 minutes | Team Interviews Patient | Group A | Group B |
| 3 | 15 minutes | Team Communication Huddle | Groups A and B |  |
| 4 | 20 minutes | Team Follow up Questions and Patient Plan | Group B | Group A |
| 5 | 10 minutes | Team Self Reflection (Group A & B) |  |  |
| 6 | 10 minutes | Standardized Patient Feedback (Group A & B) |  |  |
| SIMULATION COMPLETED | | | | |
| 7 | 25 minutes | Debrief with Faculty Facilitator (Group A & B) |  |  |
| 8 | 5-10 min. | All Teams Wrap-up |  |  |
