# Supplementary material for: Virtual Interprofessional Telehealth Education: Mixed Methods Evaluation
Source: JMIR Form Res. 2026 Aug 13;10:e92077. doi: 10.2196/92077 (PMC13472524; doi:10.2196/92077)
Supplement: Supplementary file 6 [file formative-v10-e92077-s006.docx]

**STANDARDIZED PATIENT EVALUATION CHECKLIST Frank Rivera (Scenarios 1-4)**

**Inter-professional Team: ________**

| **Collaborative Care/Patient Education Skills** | **YES** | **NO** | **N/A** | **COMMENTS** |
| --- | --- | --- | --- | --- |
| 1. The diagnosis, treatment, and importance of treatment were explained to me in plain language (no medical jargon used). |  |  |  |  |
| 2. Information was provided in small chunks and key pieces of information were repeated to me. |  |  |  |  |
| 3. I was asked about my understanding of my condition. |  |  |  |  |
| 4. I was asked about my understanding of my treatment plan (including medication, how to take them, possible side effects). |  |  |  |  |
| 5. I was asked why I thought (or did not think) taking my medication and checking my blood sugar is important. |  |  |  |  |
| 6. I was asked about my social support network |  |  |  |  |
| 7. I was asked about what potential strengths/resources I had that would help me manage this new diagnosis |  |  |  |  |
| 8. I was encouraged to ask questions and express my thoughts or concerns. |  |  |  |  |
| 9. I felt my questions were answered/ concerns were addressed. |  |  |  |  |
| 10. I felt like I was a partner in the process of my discharge. |  |  |  |  |
| **Inter-professional Teamwork and Communication Skills** | | | | |
| 11. Each team member paid attention to the information gathered by the other team members and used that information to build on without asking me to continuously repeat information. |  |  |  |  |
| 12. The team members respected each other (did not interrupt one another or contradict each other). |  |  |  |  |
| 13. Each team member introduced themselves to me. |  |  |  |  |
| 14. Each team member played a part or participated. |  |  |  |  |
| 15. The group addressed me by name or clarified my name. |  |  |  |  |
| 16. Each team member behaved warmly, but professionally throughout the encounter.   (demonstrates genuine care and concern  without losing their objectivity and ability  to deal with any aspects of your problem.  Should not remain cold and distant, nor  become overly informal and chummy. ”. |  |  |  |  |
| 17. Each team member demonstrated interest and respect towards my questions, concerns, and feelings. |  |  |  |  |
| **Had a** **Good Presence During the Telemedicine Visit**. | | | | |
| 18. The examinee **positioned** themselves **adequately** on screen. |  |  |  |  |
| 19. The examinee **made an effort** to look at the camera **to maintain eye contact**. |  |  |  |  |
| 20. The examinee was **aware of** the potential for slight **delays in videoconferencing** and  appropriately waited to continue speaking. |  |  |  |  |
| 21. **Spoke naturally** as if you were in the exam room. |  |  |  |  |
| 22. The examinee ensured a **professional environment**. |  |  |  |  |
| 23. Confirms that both parties can see and hear each other. |  |  |  |  |
| 24. Confirms patient phone number in the event of unforeseen technical difficulties. |  |  |  |  |
| 25. Confirms that the SP is in a private space and/or feels comfortable discussing personal  information. |  |  |  |  |
| **Overall** | **YES** | **NO** | **N/A** | **COMMENTS** |
| 26. I am satisfied with the care I received from this team. |  |  |  |  |
| 27. I would return to this team for future care. |  |  |  |  |
| **OVERALL COMMENTS** |  |  |  |  |
