# Supplementary material for: Virtual Interprofessional Telehealth Education: Mixed Methods Evaluation
Source: JMIR Form Res. 2026 Aug 13;10:e92077. doi: 10.2196/92077 (PMC13472524; doi:10.2196/92077)
Supplement: Supplementary file 7 [file formative-v10-e92077-s007.docx]

**FACULTY OBSERVER EVALUATION CHECKLIST Frank Rivera (Scenarios 1-4)**

| Faculty Observer: |  |
| --- | --- |
| Interprofessional Team: |  |

| **Information Sharing** | | **Action completed by** | **Comments** |
| --- | --- | --- | --- |
| 1 | Explained the condition.  (Used analogies OR mentioned problems with heart attacks, strokes, circulation, eyes, kidneys, feet, etc.) | □ Med □ OT □ PT □ RN □ SW □ Other |  |
| 3 | Emphasized to the patient the  importance of adhering to a diabetic  diet (that medicines are only part of  the management of diabetes and that  diet is a very important aspect of  controlling blood sugar. | □ Med □ OT □ PT □ RN □ SW □ Other |  |
| 4 | Emphasized to the patient he importance of checking his/her blood sugar regularly. | □ Med □ OT □ PT □ RN □ SW □ Other |  |
| 5 | Explained to the patient what normal/“well controlled” blood sugar is (normal blood sugar is 70-110) | □ Med □ OT □ PT □ RN □ SW □ Other |  |
| 6 | Explained to the patient the possible side effects from the medications (low blood sugar), how to recognize this, and what to do about it (low blood sugar) which could cause hand shaking, sweating, dizziness, hungry, irritability, fainting. | □ Med □ OT □ PT □ RN □ SW □ Other |  |
| 7 | Explained the importance of follow-up treatment and how telehealth could continue to benefit. | □ Med □ OT □ PT □ RN □ SW □ Other |  |
| 8 | Assessed the patient’s understanding of his/her diagnosis/condition. | □ Med □ OT □ PT □ RN □ SW □ Other |  |
| 9 | Assessed the patient’s understanding of the treatment (role of medications, how to take them, possible side effects). | □ Med □ OT □ PT □ RN □ SW □ Other |  |
| 10 | Assessed the patient’s understanding of self-care (how and when to check blood sugar, dietary changes to be made, checking feet, making follow-up appointments). | □ Med □ OT □ PT □ RN □ SW □ Other |  |
| 11 | Worked toward a discharge plan that addressed both the diagnosis and the patient’s concerns about his/her illness. | □ Med □ OT □ PT □ RN □ SW □ Other |  |
| 12 | Included the patient as a full partner in the discharge process. | □ Med □ OT □ PT □ RN □ SW □ Other |  |
| 15 | Engaged the patient in discussion of what may help him manage his new diagnosis | □ Med □ OT □ PT □ RN □ SW □ Other |  |
| 16 | Treats all team members respectfully. | □ Med □ OT □ PT □ RN □ SW □ Other |  |
| 17 | Considers input from all team members/communicates concerns. | □ Med □ OT □ PT □ RN □ SW □ Other |  |
| 18 | Team members actively take on roles/balanced workload. | □ Med □ OT □ PT □ RN □ SW □ Other |  |
| 19 | Responded to the patient’s unique characteristics and needs. | □ Med □ OT □ PT □ RN □ SW □ Other |  |
| 20 | Worked to establish a telehealth follow up plan | □ Med □ OT □ PT □ RN □ SW □ Other |  |

**OVERALL COMMENTS**
